# Effects of selection on production parameters and intestinal microbiota in heritage and modern broiler chickens

**DOI:** 10.1186/s40104-026-01360-8

**Published:** 2026-03-07

**Authors:** Kouassi R. Kpodo, Lori L. Schreier, Katarzyna B. Miska, Monika Proszkowiec-Weglarz

**Affiliations:** Animal Biosciences and Biotechnology Laboratory, Agricultural Research Service, United States Department of Agriculture, Beltsville, MD USA

**Keywords:** Broiler chickens, Fast growing, Genetic selection, Heritage, Slow growing

## Abstract

**Background:**

Genetic selection has improved feed efficiency and growth in broiler chickens. Intestinal microbiota plays an important role in gut functions and host metabolism; however, it is unclear whether selection for rapid growth has affected intestinal microbiota. The objective of the study was to determine the effects of genetic selection on intestinal microbiota in broiler chickens.

**Methods:**

Heritage (Athens Canadian Random Bred: ACRB), slow- and fast-growing (SG, FG) chickens were raised under similar conditions for 35 d. 16S rRNA sequencing was performed on ileal and cecal luminal (IL-L and CE-L, respectively) and mucosal bacterial populations, and data were analyzed using the qiime2 platform, differential abundance, and metabolic pathways.

**Results:**

The relative abundance of the genus *Turicibacter* was elevated (*P* < 0.05) at hatch compared to 48 h before hatch; while at species level, *Clostridium celatum* was increased (*P* < 0.05) at hatch. During the post-hatch period, body weight was higher in the SG and FG groups than in the ACRB group at each time point from d 13 to 35. In IL-L, richness (d 14) was lower (*P* < 0.05) in ACRB compared to FG and SG lines, respectively. In CE-L, richness and Shannon index were reduced (*P* < 0.05) in ACRB compared to FG lines only on d 35. The Shannon index was significantly (*P* < 0.05) lower in ACRB birds compared to FG birds on d 35. The relative abundance of genus *Escherichia* was higher (*P* < 0.05) in SG compared to other lines. FG birds were characterized by the highest (*P* < 0.05) *Streptococcus* level. Relative abundance of most of the identified species were affected only by time post-hatch and only the relative abundance of a single species *Lactobacillus salivarius* was higher (*P* < 0.05) in FG compared to ACRB birds. Predicted metabolic pathways related to biosynthesis of nucleotides and biotin, especially in the cecum, were increased in SG lines.

**Conclusion:**

These results indicate that selection for growth has affected intestinal microbiota as bacterial diversity was different in the ileum and cecum which could partly explain growth rate differences among heritage and modern lines. In addition, the increased predicted metabolic pathways in the ileum of SG birds could positively affect growth rate, and further research is needed to elucidate this hypothesis.

**Supplementary Information:**

The online version contains supplementary material available at 10.1186/s40104-026-01360-8.

## Introduction

Genetic selection has been at the forefront of the poultry industries’ success to improve feed efficiency and increase meat production over the last 60 years [1]. Several poultry companies have developed modern fast-growing broiler strains, increasing growth rates by more than 400% [2, 3]. This improvement has been partially attributed to increased digestive efficiency and a reduction in maintenance costs [3]. Selection for rapid growth has affected major organs including the intestine. Modern fast-growing birds had higher visceral organs weight [4, 5]; however, the relative weight of these organs to body weight were reduced. Genetic selection resulted in faster maturation of the intestine and increased intestinal length for all 3 segments [6] compared to heritage breeds. Selection has also reduced intestinal mucosa in relation to total body weight and increased villi surface area, which likely contributed to efficient feed utilization [6]. Intestinal length and mass are greater in fast-growing broilers compared to the unselected jungle fowl [7]. However, genetic selection to improve efficiency and meat production has inadvertently led to metabolic and morphological disorders. Studies have reported locomotion issues caused by leg deformities [8], and reduced intestinal health and function, immune response, and meat quality in fast-growing birds [1]. Another factor that may be involved in changes related to genetic selection is the composition and function of intestinal microbiota.

Intestinal microbiota has been recognized as a major player in intestinal homeostasis due to its important roles related to immune, physiology, and nutrition. Increased capacity to harvest more energy from the diet may be accomplished by the differences in intestinal microbiota among chicken lines. For example, intestinal bacteria can degrade non-digestible carbohydrates and produce short-chain fatty acids, which can add energy reserves to the host [9]. The variation in feed efficiency can be driven by intestinal microbiota, especially cecal microbiota [10] due to cecum’s prominent role in fermentation of dietary fiber [11]. It has been reported that host genetics and intestinal microbiota synergically regulate feed utilization in laying hens [12] while an association between host genetics and microbiota diversity was correlated to feed efficiency in broiler chickens [10]. This suggests that modern broiler chickens may have different microbiota profile compared to birds not selected for rapid growth. However, the interactions of intestinal microbiota with different genetic backgrounds and its influence on production performance are not well understood. Therefore, the objective of the study was to determine the effect of genetic selection on intestinal microbiota in broiler chickens. Six different lines were used in the current study, including: Athens Canadian Random Bred (ACRB) which has not undergone any selection since its establishment in 1957, Longenecker’s Heritage (LHR) and Red-Bro that have been selected for slow growth, and modern fast-growing Ross 708, Hubbard H1 (HH1), and Cobb 500 that have been intensively selected to improve feed efficiency and meat production.

## Material and methods

### Animals and experimental protocol

All animal care procedures were approved by the USDA-ARS Institutional Animal Care and Use Committee. Fertile ACRB chicken eggs were obtained from University of Georgia (Athens, GA, USA), while eggs of slow- and fast-growing broilers (SG and FG, respectively) were purchased from Longenecker’s Hatchery (Elizabethtown, PA, USA). The SG birds were represented by Hubbard RedBro and Longenecker’s Heritage Bred, while FG broilers were represented by Ross708, Cobb500 and Hubbard H1 lines. All chicken eggs (200 eggs for each line) were incubated under standard conditions at 37.5 °C and 60% relative humidity. On d 10 of incubation, non-fertilized eggs were removed from the incubator after candling. The fertility was calculated based on the number of empty eggs for each line as described previously [13]. After hatch, hatchlings ACRB – 131, SG – 282 (140 for Hubbard RedBro and 142 for Longenecker’s Heritage), and FG – 414 (152 for Ross708, 138 for Cobb500, and 124 for Hubbard H1) were evenly distributed among 24 floor pens. Each pen was equipped with a heating lamp, feeder and nipple drinker, and the floor was covered with wood shavings. Regardless of the line, chicks had ad libitum access to the same commercial type corn-soybean meal-based starter (hatch to d 21) or grower (d 21–35) diets that were formulated to meet or exceed NRC [14] specifications. There were 4 pens with ACRB chicks, 8 pens with SG birds and 12 pens with FG birds. Contrary to FG and SG, there is only one line for the heritage birds ACRB. This strain was created in 1957 by scientists through random breeding of commercial meat-type chickens. It has not undergone any selection since then and has been extensively used as a control to study changes in commercial broilers over time [15]. The breeding stock has been maintained at the University of Georgia, Athens, GA, USA.

### Tissue sampling

Tissue samples (*n* = 4 for ACRB, *n* = 8 for SG, and *n* = 12 for FG) were collected at each of the following timepoints: 48 h before hatch (e19), at hatch, and d 7, 14, 21, 28 and 35 post-hatch. Body weight and feed intake were determined for each pen at each sampling time point. Due to the small size of the embryos and hatchlings, ileal (IL) and cecal (CE) samples were collected from two embryos or hatchlings and pooled together. To determine ileal and cecal luminal (IL-L and CE-L, respectively) and mucosal (IL-M and CE-M, respectively) bacterial populations, the distal part of the ileum and one cecum content as well as their corresponding epithelial scrapings were collected and snap-frozen in liquid nitrogen. Samples were stored at −80 °C until bacterial DNA isolation.

### DNA isolation and library preparation

DNA was extracted from ileal and cecal contents and scrapings using a DNeasy PowerSoil kit (Qiagen, Valencia, CA, USA) and QIAcube instrument (Qiagen) according to the manufacturer’s protocol. Isolated DNA concentration and quality were evaluated using NanoDrop (TermoFisher Scientific, Inc., Waltham, MA, USA) and Tapestation System (Agilent Technologies, Santa Clara, CA, USA), respectively. The 16S rRNA gene amplicon libraries were generated using the workflow and chemistry supplied by Illumina (Illumina, Inc., San Diego, CA, USA) and PCR primers (Forward: 5′-TCGTCGGCAGCGTCAGATGTGTATAAGAGACAGCCTACGGGNGGCWGCAG-3′ and Reverse: 5′-GTCTCGTGGGCTCGGAGATGTGTATAAGAGACAGGACTACHVGGGTATCTAATCC-3′) targeted the V3–V4 variable region of the 16S gene. Amplicon PCR followed by index PCR and PCR amplicon cleaning were performed as described previously [16]. Concentration and quality of the amplicons were determined using Qubit 3 (Thermo Fisher Scientific) and Bioanalyzer (Agilent Technologies), respectively. The pooled (96 barcoded amplicons) DNA library (4 nmol/L) and PhiX control v3 (Illumina, Inc., 4 nmol/L) were denatured with 0.2 mol/L NaOH (Sigma-Aldrich, Corp., St. Louis, MO, USA) and diluted to a final concentration of 4 pmol/L. The library was mixed with PhiX control (20% v/v) and pair-end 2 × 300-bp sequencing was performed using the Illumina MiSeq platform and a MiSeq Reagent Kit v3 (Illumina). The 16S rRNA gene sequences determined in this study were deposited in the NCBI Sequence Read Archive (SRA) database (SRA PRJNA1280974).

### 16S rRNA gene sequence, data processing and analysis

Quantitative Insight Into Microbial Ecology (QIIME) software package 2 (version 2021.4.0, https://qiime2.org) was used to perform quality control and analysis of the sequence reads [17]. Raw Fastq files were demultiplexed using q2-demux and quality filtered and dereplicated with q2-dada2 [18]. Sequences with an average Phred score lower than 25 were removed. MAFFT was used for multiple sequence alignment [19]. FastTree was used to generate phylogenetic trees [20]. Amplicon sequence variants (ASVs) from DADA2 were assigned taxonomy via the q2-feature-classifier classify-sklearn naïve Bayes taxonomy classifier [21] using SILVA version 138 99% operational taxonomic unit reference sequences and taxonomy [22]. The SILVA reference sequences and taxonomy files were pre-formatted using RESCRIPt (obtained at https://library.qiime2.org/data-resources#qiime-2-2021-4-2024-2), a process used to reduce inconsistencies and improve processing by removing duplicate sequences that are assigned different taxonomies [23].

Data were rarefied to a sequencing depth where the bacterial diversity of the remaining samples was well represented (Table 1) for calculation of alpha and beta diversities. Alpha diversity indices (ASVs, Shannon’s diversity index, Pielou’s Evenness, and Faith’s Phylogenetic Diversity) were obtained through the QIIME 2 package. Alpha diversity metrics were used to measure species richness and/or evenness within one sample, and the non-parametric Kruskal–Wallis test was used to analyze differences in alpha diversity between time points, broiler lines, and time × line interaction. Analysis of beta diversity based on Unweighted UniFrac was performed in QIIME2. To test for significance in UniFrac distances, the non-parametric permutational analysis of variance (PERMANOVA) test was used. Principal coordinates analysis (PCoA) was used to visualize distances between treatment groups as well as to visualize clustering of samples (QIIME2) [24]. PCoA graphs were created using Qiime2R [25] and ggplot2 [26].

**Table 1:** Sequencing information for ileal (IL) and cecal (CE) luminal (L) and mucosal (M) samples

Item	IL^1^	CE^1^	IL-L^2^	IL-M^2^	CE-L^2^	CE-M^2^
Sample number	48	48	120	120	120	120
Raw reads						
Total	856,369	823,730	16,736,616	8,626,376	13,389,721	13,326,299
Mean	17,841	17,161	139,471	71,886	111,581	111,052
Reads after quality trimming						
Total	166,307	160,944	8,074,168	5,156,013	5,988,563	5,294,482
Mean	3,464	5,345	67,284	42,966	49,904	44,120
Sequencing depth for analysis	947	632	11,348	3,810	11,178	7,811

^1^Samples collected from −48 h to hatch

^2^Samples collected from 7 to 35 d post-hatch

Relative abundance data for microbiota composition were obtained by normalization to the number of reads per sample. The data were analyzed with non-parametric Scheirer-Ray-Hare test [27], followed by post-hoc Dunn test [28] with *P*-values adjusted for multiple comparisons using Benjamini–Hochberg method [29] in R [26]. Significance was set at *P* < 0.05.

QIIME data were transformed using R package Compositions [26], and Phylogenetic Investigation of Communities by Reconstruction of Unobserved States (PICRUSt)2 [30] was used to predict metagenome pathways for each primer set using the MetaCyc database (MetaCyc.org) [31]. Statistical Analysis of Metagenomic Profiles (STAMP 2.1.3) [32] was used to create a visualization of metabolic pathway comparison. Within STAMP software, a two group comparison was performed using Welch *t*-test [33] corrected for false discovery rate (FDR, Benjamini–Hochberg analysis [29]).

Linear discriminant analysis (LDA) Effect Size (LEfSe) algorithm [34] was used to identify taxa with significant differential abundance between treatment groups. LEfSe graphs were created using ggplot2 [26]. For the LEfSe analysis, alpha value of 0.05 for Kruskal–Wallis test was used, and the threshold for the log_10_LDA score was set at 2.0.

### Statistical analysis

Body weight, feed intake, and feed conversion ratio data were considered as repeated measures and analyzed by two-way ANOVA using GLIMMIX procedure of the Statistical Analysis System (SAS) software, version 9.4 (SAS Institute, Cary, NC, USA). The pen was considered an experimental unit. Data from individual lines were pooled into ACRB, FG and SG for statistical analysis. Least significant differences were used to determine which means differed significantly with Tukey-Krammer adjustment for multiple comparisons. Spearman correlation analysis between body weight and relative abundance of taxonomic groups at genus level was performed in SAS software. Significance was set at *P* < 0.05 for all analysis.

## Results

### Performance

**Fig. 1:**
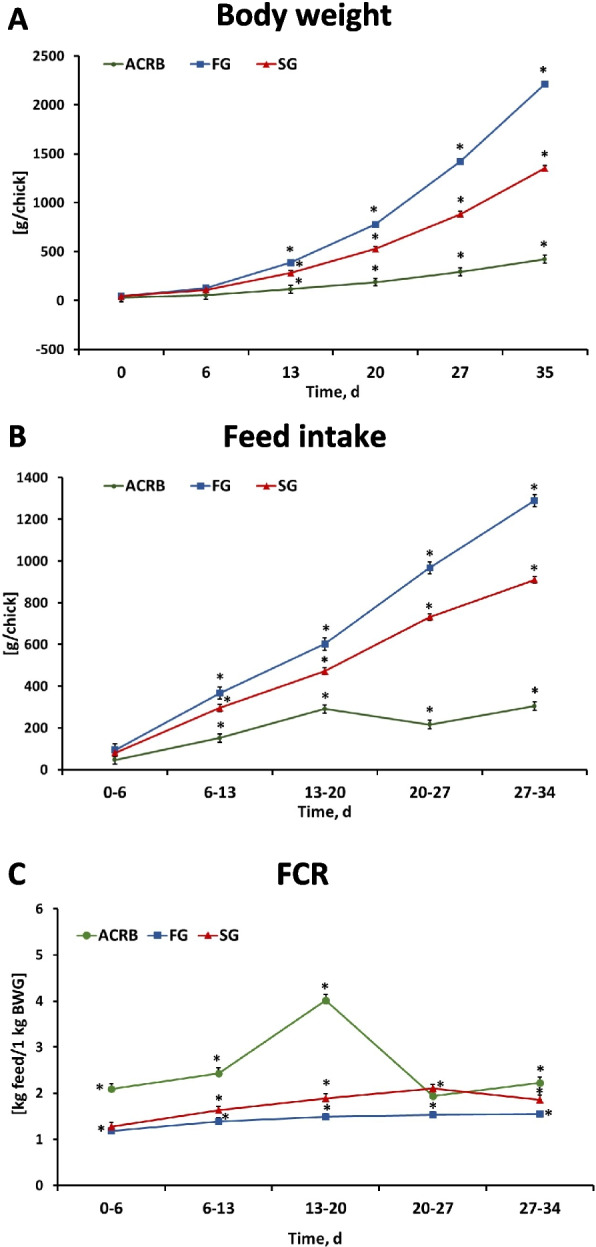
Effects of line on (**A**) body weight, (**B**) feed intake, and (**C**) feed conversion ratio (FCR) from hatch (d 0) until d 35. Each value represents the mean ± SE of 4–12 pens. Asterisks denote significant (*P* < 0.05) differences among lines. ACRB: Athens Canadian Random Bred; FG: Fast growing chickens; SG: Slow growing chickens

The fertility was 81.03%, 89.84% and 90.95% for ACRB, FG and SG, respectively. The hatchability rate ranged from 77.7% for ACRB to 91.25% and 92.46% for FG and SG, respectively. There were no significant (*P* > 0.05) differences in body weight between SG, FG, and ACRB chicks at hatch and 6 d post-hatch; however, significant differences in body weight between SG and FG, as well as between SG, FG and ACRB were observed from d 13 onward until the end of the experiment at d 35 (Fig. 1A). From d 13, FG birds were characterized by the highest body weight while ACRB had the lowest body weight overall.

Similar to body weight, there were significant (*P* < 0.05) differences in feed intake among lines starting from d 6 post-hatch, with the highest feed intake observed in FG birds, followed by SG lines and the lowest one in ACRB birds (Fig. 1B). Feed conversion ratio (FCR) was greater in ACRB compared to both FG and SG on d 6 post-hatch; however, it remained significantly different among lines, with highest FCR observed in ACRB followed by SG and FG from d 13 to 34 except on d 27 where ACRB was intermediate (Fig. 1C). The individual lines’ body weight, feed intake, and FCR were reported previously [35].

### Sequencing information

The sample number, total and average number of reads per tissue before and after quality trimming are presented in Table 1. Samples collected from embryos (−48 h, e19) and at hatch were characterized by lower number of reads in comparison to samples collected later (from 7 d post-hatch onward), therefore these samples (−48 h and hatch) were analyzed separately.

### Microbiota of embryos and hatchlings

**Fig. 2:**
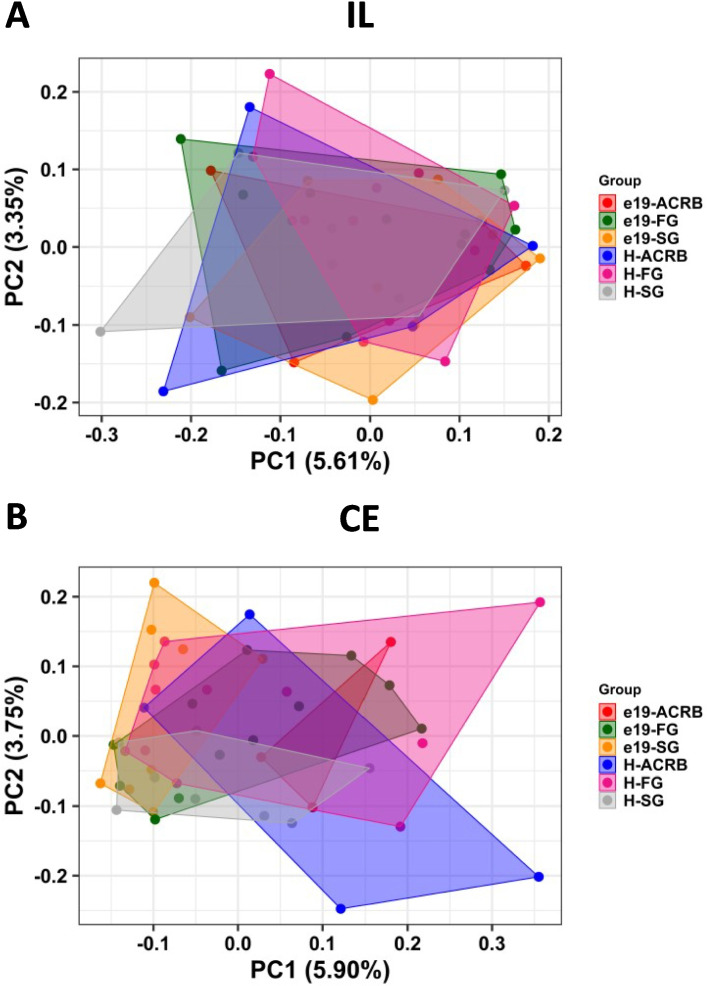
Effects of line on beta diversity of (**A**) ileal (IL) and (**B**) cecal (CE) bacterial population in embryos (−48 h, e19) and chicks at hatch (H) using the principal coordinate analysis (PCoA) based on the Unweighted UniFrac distances between specific groups. ACRB: Athens Canadian Random Bred; FG: Fast growing chickens; SG: Slow growing chickens

The alpha diversity indexes (number of ASVs, richness, evenness and Shannon index) in ileal and cecal samples collected from embryos 48 h before hatch and at hatch were similar (*P* > 0.05) regardless of the chicken line (FG, SG and ACRB) (Table 2). Principal coordinate analysis (PCoA) shows overlapping clustering of FG, SG and ACRB samples at −48 h and at hatch in ileum (Fig. 2A). More distinct clustering was observed in CE samples at the same time points (Fig. 2B). Results from PERMANOVA analysis are presented in Table 3, with no significant (*P* > 0.05) differences in IL due to time (−48 h vs. hatch) or line (FG vs. SG vs. ACRB) or their interaction, while there was a significant (*P* < 0.05) line and Time × Line effect observed in CE.

**Table 2:** Kruskal–Wallis *P*-values for interactive Time × Line or main (Time, Line) effects for alpha diversity indices in ileal (IL) and cecal (CE) samples

Item	Pr = F	
	IL	CE
ASV		
Time	0.915	0.826
Line	0.953	0.113
Time × Line	0.999	0.377
Shannon diversity index		
Time	0.932	0.921
Line	0.991	0.157
Time × Line	0.999	0.432
Faith’s Phylogenetic Diversity (Richness) index		
Time	0.888	0.448
Line	0.974	0.098
Time × Line	0.998	0.282
Pielou’s Evenness index		
Time	0.482	0.276
Line	0.062	0.896
Time × Line	0.284	0.254

Samples were collected from −48 h post-hatch and at hatch

*ASV* Amplicon sequence variant

**Table 3:** PERMANOVA *P*-values for interactive (Time × Line) or main (Time, Line) effects for bacterial community beta diversity (PERMANOVA results) in ileal (IL) and cecal (CE) samples

Item	Pr = F	
	IL	CE
Unweighted UniFrac Distance		
Time	0.348	0.695
Line	0.324	0.002
Time × Line	0.376	0.024

Samples were collected from −48 h post-hatch and at hatch

Taxonomic composition of the IL and CE in ACRB, FG and SG birds is presented in Fig. S1. Overall, most of the bacterial reads (from 60% to 98%) were identified as unclassified bacteria (UNCL) in IL and CE. Early ileal microbiota was also composed of *SMB53*, *Delftia*, *Turicibacter*, *Lactobacillus* and low abundance reads (LAR) that represent bacterial taxa characterized by very low abundance at genus level (Fig. S1A) and *Lysinibacillus boronitolerans*, *Clostridium celatum*, *Escherichia coli* and LAR at species level (Fig. S1C). The relative abundance of *Turicibacter* was significantly elevated at hatch in comparison to e19 (48 before hatch) at genus level (Fig. S1B) while at species level, chicks at hatch were characterized by higher (*P* < 0.05) relative abundance of *Clostridium celatum* and LAR (Fig. S1D and E, respectively). In CE, bacterial reads could not be classified at family level (Fig. S1F and G). At phylum level, cecal microbiota was composed mainly of UNCL, followed by OD1, Proteobacteria and LAR while at family level, UNCL and LAR were detectable (Fig. S1F and G, respectively). No significant (*P* > 0.05) effect of time (48 before hatch vs. hatch) or line (FG, SG or ACRB) on microbiota composition of CE were found (data not presented). Differentially abundant taxa were detected using LEfSe analysis only in IL between 48 before hatch and at hatch samples (Fig. S1H) with more taxa abundant in hatchings in comparison to 48 h before hatch.

### Alpha diversity in luminal and mucosal bacterial populations in post-hatch chickens

**Fig. 3:**
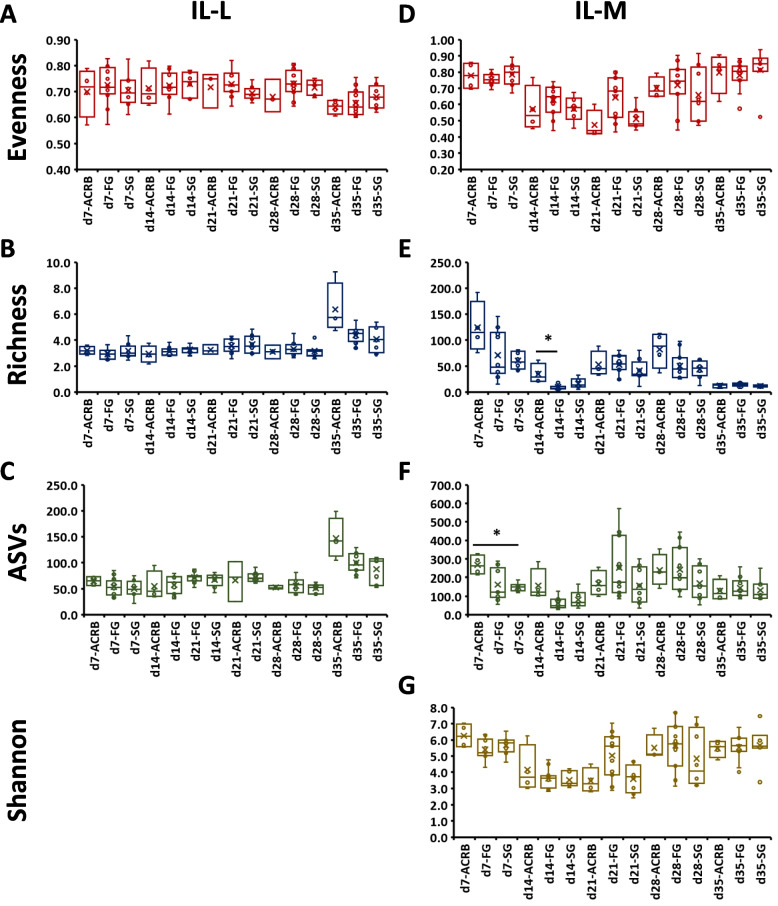
Effects of line and time on alpha diversity indices (**A**, **D**) Evenness, (**B**, **E**) Richness, (**C**, **F**) number of amplicon sequence variants (ASV), and (**G**) Shannon index in ileal luminal (IL-L) and mucosal (IL-M) bacterial population from d 7 through d 35 post-hatch. When the interaction between time and treatment was significant, only significant (*P* < 0.05) differences between line at single time are shown as indicated by asterisk. ACRB: Athens Canadian Random Bred, FG: Fast growing chickens, SG: Slow growing chickens

From d 7 of post-hatch development, ileal and cecal samples were collected and analyzed for luminal and mucosal bacterial population. The effects of sampling time and line interaction on alpha diversity indices in ileal luminal (IL-L) and mucosal (IL-M) bacterial populations are shown in Fig. 3. In IL-L, alpha diversity indices such as evenness (Fig. 3A), richness (Fig. 3B), and number of ASVs (Fig. 3C) were mainly affected (*P* < 0.05) by time × line interaction that was driven by time of sampling (Table 4) without any significant differences between lines at each time point. Shannon index was similar between samples regardless of time or line (data not shown). No effect of line (ACRB, FG or SG) was observed in IL-L (Table 4).

**Table 4:** Kruskal–Wallis *P*-values for interactive (Time × Line) or main (Time, Line) effects for alpha diversity indices in ileal (IL) and cecal (CE) luminal (L) and mucosal (M) samples

Item	Pr = F			
	IL-L	IL-M	CE-L	CE-M
ASV				
Time	< 0.001	< 0.001	< 0.001	< 0.001
Line	0.891	0.139	0.588	0.319
Time × Line	< 0.001	< 0.001	< 0.001	< 0.001
Shannon diversity index				
Time	0.059	< 0.001	< 0.001	< 0.001
Line	0.779	0.526	0.542	0.297
Time × Line	0.231	< 0.001	< 0.001	< 0.001
Faith’s Phylogenetic Diversity (Richness) index				
Time	< 0.001	< 0.001	< 0.0001	< 0.001
Line	0.994	0.104	0.468	0.163
Time × Line	< 0.001	< 0.001	< 0.001	0.003
Pielou’s Evenness index				
Time	0.001	< 0.001	< 0.001	< 0.001
Line	0.267	0.560	0.884	0.156
Time × Line	0.033	< 0.001	< 0.001	< 0.001

Samples were collected from d 7 to 35 post-hatch

*ASV* Amplicon sequence variant

Alpha diversity in IL-M (Fig. 3D–G) was characterized by significant changes due to time of sampling (Table 4) and the differences between lines at each time point were significant, but the significance depended on the time of sampling for richness (Fig. 3E) and number of ASVs (Fig. 3F). Richness was higher (*P* < 0.05) in ACRB compared to FG birds on d 14 (Fig. 3E) while ACRB birds had higher (*P* < 0.05) number of ASVs in comparison to SG birds at d 7 (Fig. 3F). No significant (*P* > 0.05) pairwise comparison between lines at each time point was found for evenness and Shannon index in IL-M (Fig. 3D and G).

**Fig. 4:**
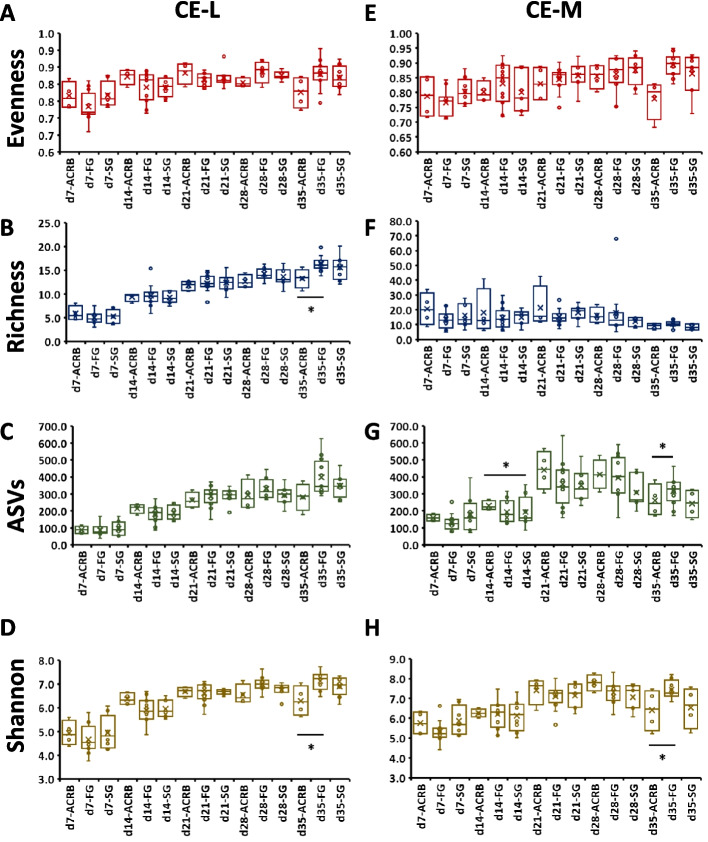
Effects of line and time on alpha diversity indices (**A**, **E**) Evenness, (**B**, **F**) Richness, (**C**, **G**) number of amplicon sequence variants (ASV), and (**D**, **H**) Shannon index in cecal luminal (CE-L) and mucosal (CE-M) bacterial population from d 7 through d 35 post-hatch. When the interaction between time and treatment was significant, only significant (*P* < 0.05) differences between lines at single age are shown as indicated by asterisk. ACRB: Athens Canadian Random Bred; FG: Fast growing chickens; SG: Slow growing chickens

In cecal samples, CE-L and CE-M (Fig. 4) there were time by line interactions mainly due to time of sampling (Table 4). In CE-L, ACRB birds had lower (*P* < 0.05) richness and Shannon index in comparison to FG birds, but only on d 35 (Fig. 4B and D). In CE-M, the ASV numbers were significantly reduced between ACRB and SG birds on d 14 and ACRB and FG birds on d 35 (Fig. 4G). Shannon index was significantly (*P* < 0.05) lower in ACRB birds in comparison to FG birds on d 35 (Fig. 4H). Similar to IL samples, no line effects were observed in CE samples (Table 4).

### Beta diversity in luminal and mucosal bacterial populations in post-hatch chickens

**Fig. 5:**
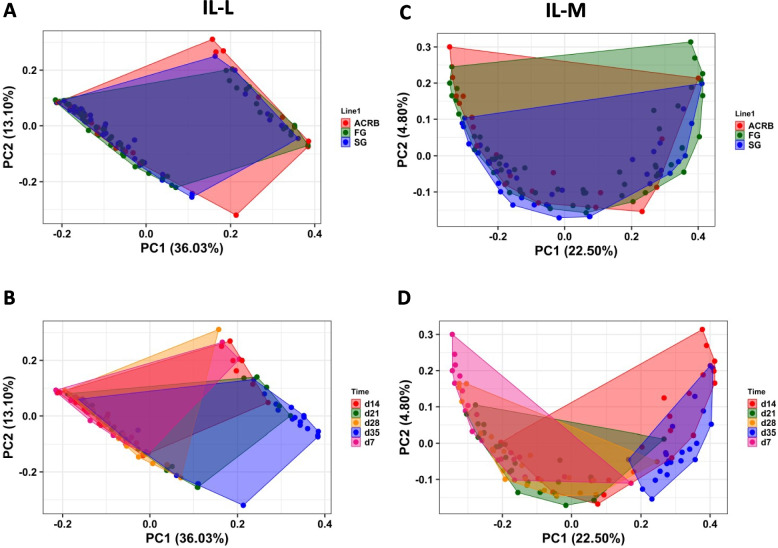
Effects of line (**A**, **C**) and time (**B**, **D**) on beta diversity of ileal luminal (IL-L) and mucosal (IL-M) bacterial population from d 7 through d 35 post-hatch using the principal coordinate analysis (PCoA) based on the Unweighted UniFrac distances between specific groups. ACRB: Athens Canadian Random Bred; FG: Fast growing chickens; SG: Slow growing chickens

**Fig. 6:**
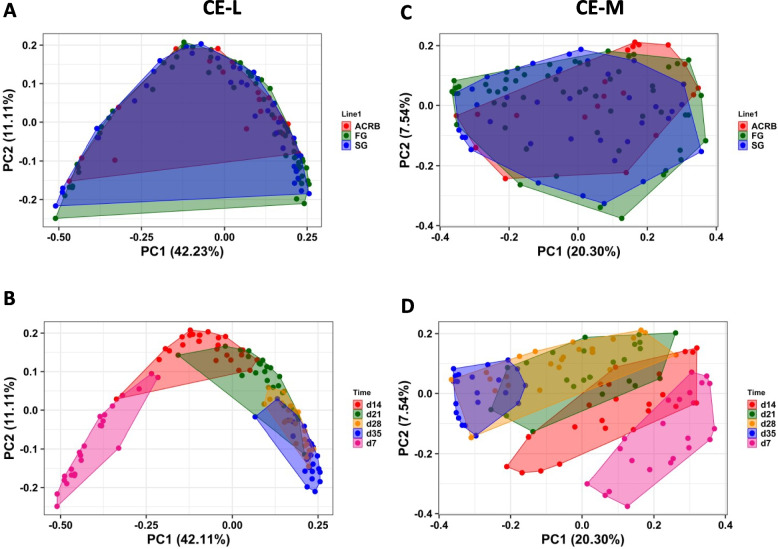
Effects of (**A**, **C**) line and (**B**, **D**) time on beta diversity of cecal luminal (CE-L) and mucosal (CE-M) bacterial population from d 7 through d 35 post-hatch using the principal coordinate analysis (PCoA) based on the Unweighted UniFrac distances between specific groups. ACRB: Athens Canadian Random Bred; FG: Fast growing chickens; SG: Slow growing chickens

PERMANOVA analysis based on Unweighted UniFrac distance matrix was performed to determine similarities between bacterial communities (Table S1). Bacterial population in IL-L, IL-M, CE-L, and CE-M were affected by time and time by line interaction, and only beta diversity of Il-L microbiota was affected by line (Table S1). Figure 5 shows PCoA based on Unweighted UniFrac distance matrix in Il-L and IL-M microbiota showing line and time effects. No differences in beta diversity between ACRB, FG and SG were observed (Fig. 5A and C), while a shift in sample clustering was observed in IL-L and IL-M over time (Fig. 5B and D). Similar results were observed in CE-L and CE-M (Fig. 6). The clustering of samples by line in both CE-L and CE-M overlapped (Fig. 6A and C, respectively), while the clustering by time showed distinctive pattern in CE-L and CE-M (Fig. 6B and D).

### Taxonomic composition

**Fig. 7:**
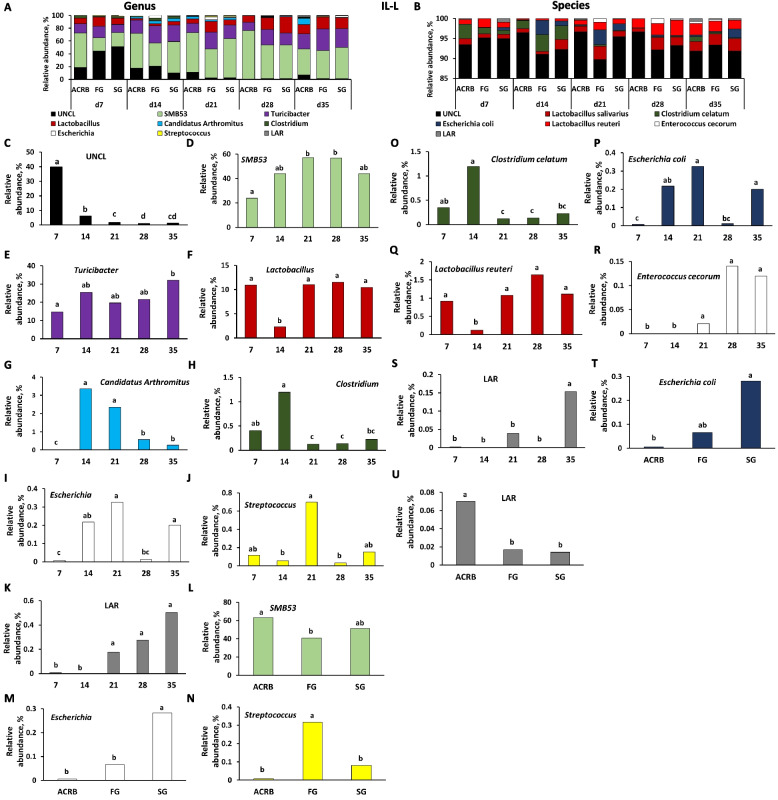
Effects of line and time on relative bacterial abundance (%) in ileal luminal (IL-L) bacterial populations from d 7 through d 35 post-hatch at genus and species level. Taxonomic profile of chicken IL-L at (**A**) genus and (**B**) species level. Effects of line or time on (**C**) unclassified bacteria reads (UNCL), (**D**, **L**) *SMB53*, (**E**) *Turicibacter*, (**F**) *Lactobacillus*, (**G**) *Candidatus Arthromitus*, (**H**) *Clostridium*, (**I**, **M**) *Escherichia*, (**J**, **N**) *Streptococcus*, and (**K**) low abundance reads (LAR) on genus level, and (**O**) *Clostridium celatum*, (**P**, **T**) *Escherichia coli*, (**Q**) *Lactobacillus reuteri*, (**R**) *Enterococcus cecorum*, and (**S**, **U**) LAR on species level. Different characters denote statistically significant (*P* < 0.05) differences between lines (ACRB, FG, SG) or times (d 7, 14, 21, 28, and 35). ACRB: Athens Canadian Random Bred; FG: Fast growing chickens; SG: Slow growing chickens

**Fig. 8:**
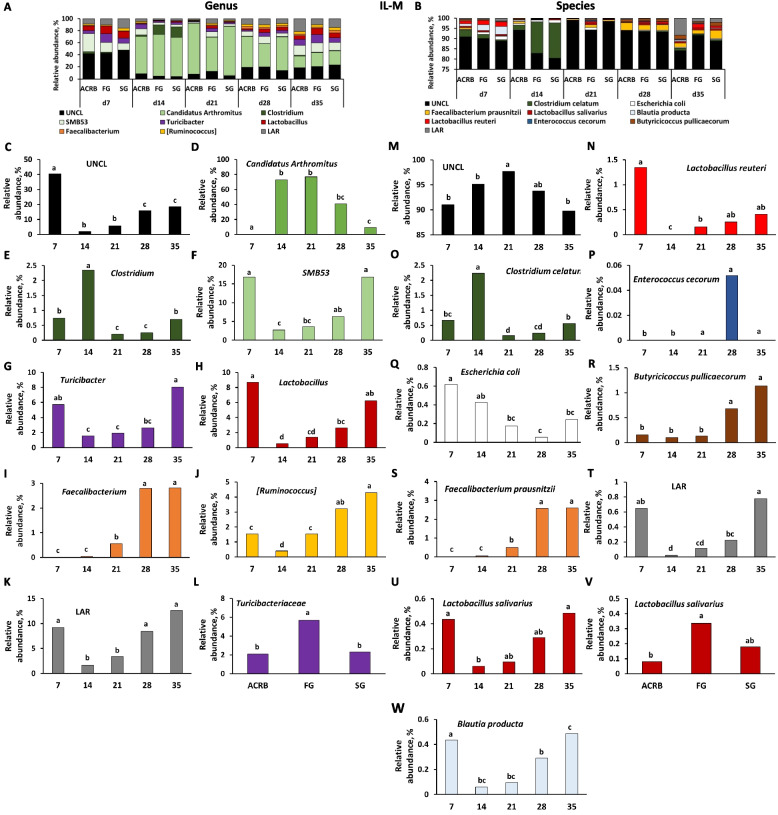
Effects of line and time on relative bacterial abundance (%) in ileal mucosal (IL-M) bacterial populations from d 7 through d 35 post-hatch at genus and species level. Taxonomic profile of chicken IL-M at (**A**) genus and (**B**) species level. Effect of line or time on (**C**) unclassified bacteria reads (UNCL), (**D**) *Candidatus Arthromitus*, (**E**) *Clostridium*, (**F**) *SMB53*, (**G**, **L**) *Turicibacter*, (**H**) *Lactobacillus*, (**I**) *Faecalibacterium*, (**J**) [*Ruminococcus*], and (**K**) low abundance reads (LAR) on genus level, and (**M**) UNCL, (**N**) *Lactobacillus reuteri*, (**O**) *Clostridium celatum*, (**P**) *Enterococcus cecorum*, (**Q**) *Escherichia coli*, (**R**) *Butyricicoccus pullicaececorum*, (**S**) *Faecallibacterium prausnitzii*, (**T**) LAR, (**U**, **V**) *Lactobacillus sallivarius*, and (**W**) *Blautia producta* on species level. Different characters denote statistically significant (*P* < 0.05) differences between lines (ACRB, FG, SG) or times (d 7, 14, 21, 28, and 35). ACRB: Athens Canadian Random Bred; FG: Fast growing chickens; SG: Slow growing chickens

**Fig. 9:**
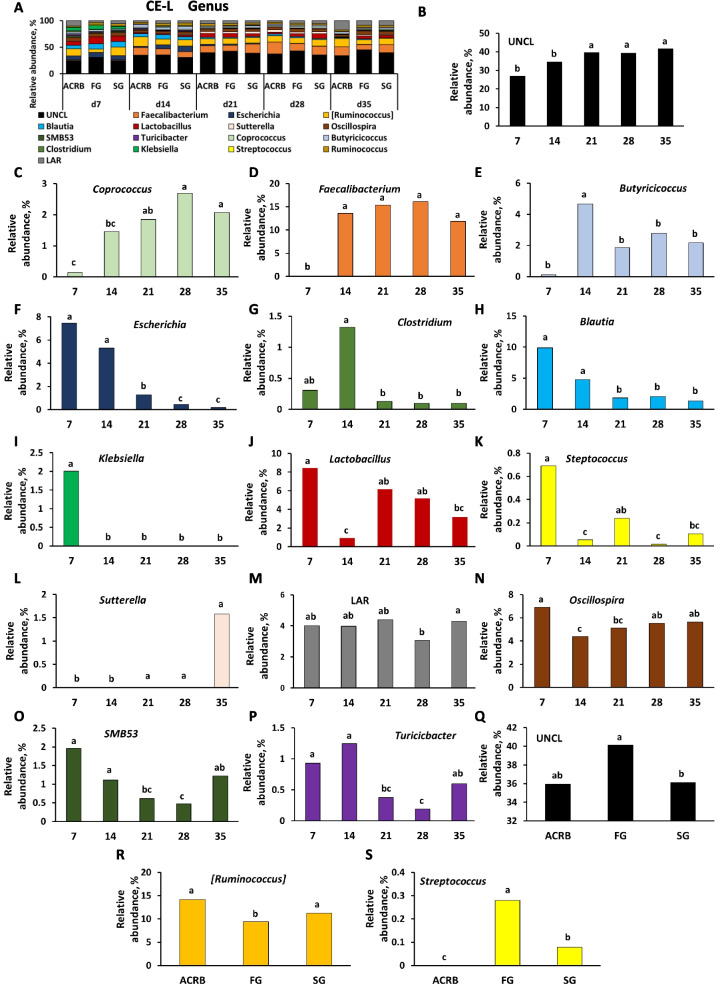
Effects of line and time on relative bacterial abundance (%) in cecal luminal (CE-L) bacterial populations from d 7 through d 35 post-hatch at genus level. Taxonomic profile of chicken CE-L at (**A**) genus level. Effect of line or time on (**B**, **Q**) unclassified bacteria reads (UNCL), (**C**) *Coprococcus,* (**D**) *Faecalibacterium*, (**E**) *Butyricicoccus*, (**F**) *Escherichia,* (**G***) Clostridium*, (**H**) *Blautia,* (**I**) *Klebsiella*, (**J**) *Lactobacillus*, (**K**, **S**) *Streptococcus*, (**L**) *Sutterella*, (**M**) low abundance reads (LAR), (**N**) *Oscillospira*, (**O**) *SMB53*, (**P**) *Turicibacter*, (**R**) [*Ruminococcus*]. Different characters denote statistically significant (*P* < 0.05) differences between lines (ACRB, FG, SG) or times (d 7, 14, 21, 28, and 35). ACRB: Athens Canadian Random Bred; FG: Fast growing chickens; SG: Slow growing chickens

**Fig. 10:**
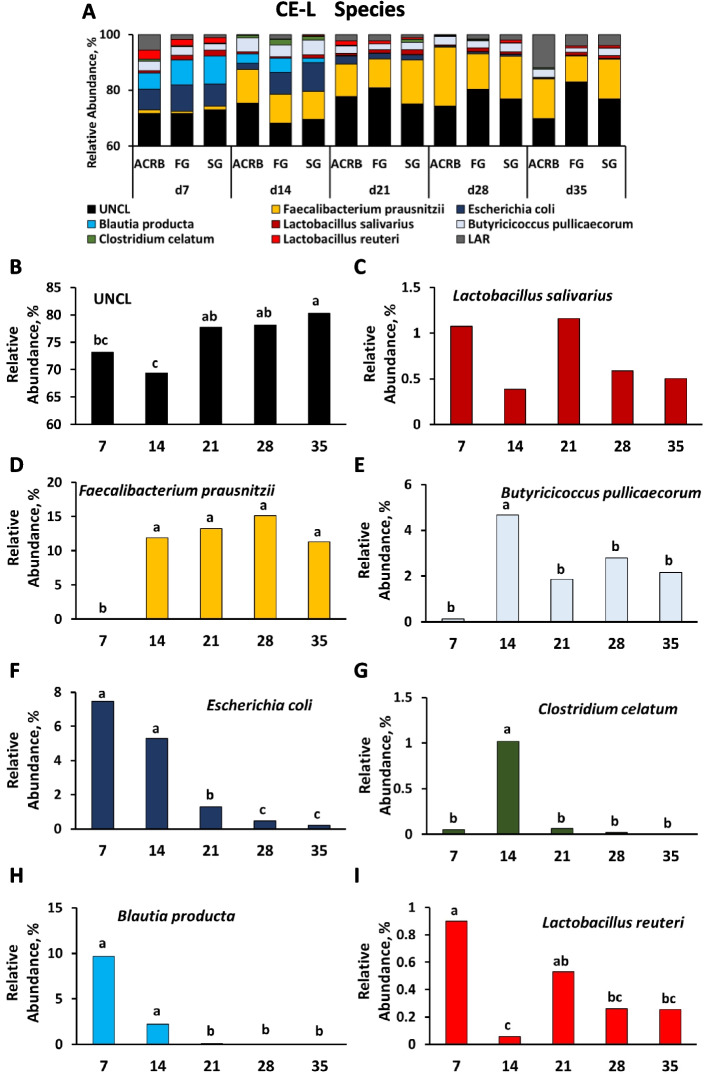
Effects of line and time on relative bacterial abundance (%) in cecal luminal (CE-L) bacterial populations from d 7 through d 35 post-hatch at species level. Taxonomic profile of chicken CE-L at (**A**) species level. Effects of line or time on (**B**) UNCL, (**C**) *Lactobacillus sallivarius*, (**D**) *Faecallibacterium prausnitzii*, (**E**) *Butyricicoccus pullicaececorum,* (**F**) *Escherichia coli*, (**G**) *Clostridium celatum*, (**H**) *Blautia producta*, and (**I**) *Lactobacillus reuteri*. Different characters denote statistically significant (*P* < 0.05) differences between lines (ACRB, FG, SG) or times (d 7, 14, 21, 28, and 35). ACRB: Athens Canadian Random Bred; FG: Fast growing chickens; SG: Slow growing chickens

**Fig. 11:**
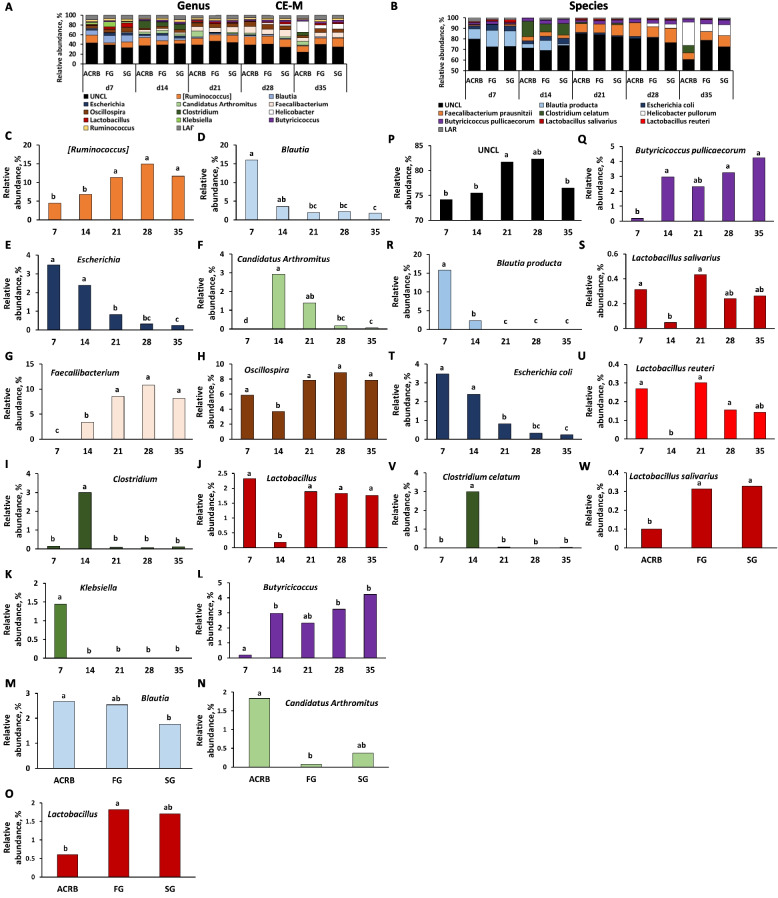
Effects of line and time on relative bacterial abundance (%) in cecal mucosal (CE-M) bacterial populations from d 7 through d 35 post-hatch at genus and species level. Taxonomic profile of chicken CE-M at (**A**) genus and (**B**) species level. Effect of line or time on (**C**) [*Ruminococcus*], (**D**) *Blautia,* (**E**) *Escherichia,* (**F**) *Candidatus Arthromitus*, (**G**) *Faecalibacterium*, (**H**) *Oscillospira*, (**I**) *Clostridium*, (**J**, **O**) *Lactobacillus*, (**K**) *Klebsiella*, (**L**) *Butyricicoccus*, (**M**) *Blautia*, and (**N**) *Candidatus Arthromitus* on genus level, and (**P**) unclassified bacteria reads (UNCL), (**Q**) *Butyricicoccus pullicaececorum*, (**R**) *Blautia producta,* (**S**, **W**) *Lactobacillus salivarius*, (**T**) *Escherichia coli*, (**U**) *Lactobacillus reuteri*, and (**V**) *Clostridium celatum* on species level. Different characters denote statistically significant (*P* < 0.05) differences between line (ACRB, FG, SG) or times (d 7, 14, 21, 28, and 35). ACRB: Athens Canadian Random Bred; FG: Fast growing chickens; SG: Slow growing chickens

Figures 7, 8, 9, 10, and 11 present the taxonomic composition of bacterial communities for IL-L, IL-M, CE-L, and CE-M, respectively, at genus and species levels. For all five figures, the top panel (A for genus level and B for species level) shows the overall taxonomic composition of microbiota, while panels below reveal the significant (*P* < 0.05) changes due to time and/or line of birds for individual bacterial taxa (Figs. 7–11).

Relative abundance of nine genera was significantly (*P* < 0.05) affected by time of sampling including UNCL (Fig. 7C), *SMB53* (Fig. 7D), *Turicibacter* (Fig. 7E), *Lactobacillus* (Fig. 7F), *Candidatus Arthromitus* (Fig. 7G), *Clostridium* (Fig. 7H), *Escherichia* (Fig. 7I), *Streptococcus* (Fig. 7J) and LAR (Fig. 7K) showing different pattern of changes. Only three genera were affected by chicken line (Fig. 7L–N). FG birds had a lower relative abundance of *SMB53* in comparison to ACRB birds (Fig. 7L), *Escherichia* relative abundance was significantly (*P* < 0.05) higher in SG birds in comparison to other lines (Fig. 7M), and FG birds were characterized by the highest (*P* < 0.05) *Streptococcus* level (Fig. 7N). Similar to genus level, identified species including *Clostridium celatum*, *Escherichia coli*, *Lactobacillus reuteri, Enterococcus cecorum* and LAR were affected by time (Fig. 7O–S) while only *Escherichia coli* (Fig. 7T) and LAR (Fig. 7U) relative abundance was affected by line with ACRB birds characterized by lower (*P* < 0.05) *E. coli* level than SG birds and the highest (*P* < 0.05) amount of LAR in comparison to FG and SG birds in IL-L.

Taxonomic composition and changes in bacterial abundance due to time and line in IL-M are presented in Fig. 8. At genus level, except for Turicibacteriaceae (Fig. 8L), relative abundance of UNCL, *C*. *Arthromitus, Clostridium*,* SBM53*,* Turicibacter*,* Lactobacillus*,* Faecallibacterum*, [*Ruminococcus*], and LAR was affected (*P* < 0.05) by time of sampling post-hatch (Fig. 8C–K). FG birds were characterized by the highest (*P* < 0.05) relative abundance of Turicibacteriaceae in comparison to ACRB and SG birds (Fig. 8L). Similarly, at the species level, identified species were affected only by time post-hatch (Fig. 8M–U and W) and include UNCL, *L. reuteri, C. celatum, E. cecorum*, *E. coli*, *Butyricicoccum pullicaecorum*, *Faecalibacterium prausnitzii*, LAR, *L. salivarius*, and *Blautia producta*. Only the relative abundance of *Lactobacillus salivarius* was affected by line with FG birds characterized by higher (*P* < 0.05) level of *L. salivarius* in comparison to ACRB birds (Fig. 8V).

### Differential bacterial abundance between lines of chickens

Changes in relative abundance of bacterial genera in CE-L are depicted in Fig. 9A–P. Fifteen genera were significantly (*P* < 0.05) affected only by time post-hatch (Fig. 9B–P). Only UNCL, [*Ruminococcus*], and *Streptococcus* were affected by line of birds. The relative abundance of UNCL was greater in FG compared to SG (Fig. 9Q). In the case of [*Ruminococcus*], FG birds had significantly (*P* < 0.05) lower relative abundance in comparison to ACRB and SG birds (Fig. 9R), while relative abundance of *Streptococcus* was the highest (*P* < 0.05) in FG birds followed by SG and ACRB birds (Fig. 9S). On the species level, relative abundance of UNCL, (Fig. 10B), *Lactobacillus salivarius* (Fig. 10C), *Faecalibacterium prausnitzii* (Fig. 10D), *Butyricicoccum pullicaecorum* (Fig. 10E), *E. coli* (Fig. 10F), *Clostridium celatum* (Fig. 10G)*, Blautia producta* (Fig. 10H)*,* and *L. reuteri* (Fig. 10I) were only affected (*P* < 0.05) by time post-hatch, but not by line of chickens.

In CE-M, ten bacteria at genus level (Fig. 11A, C–L) and seven at species level showed significant changes in relative abundance during post-hatch development, while only three genera (Fig. 11M–O) and one species (Fig. 11W) were affected by line. ACRB birds were characterized by significantly (*P* < 0.05) higher relative abundance of *Blautia* (Fig. 11M) in comparison to SG and *Candidatus Arthromitus* (Fig. 11N) in comparison to FG birds, while *Lactobacillus* genus abundance (Fig. 11O) was lower (*P* < 0.05) in the ACRB group than in the FG group. *Lactobacillus salivarius* species were lower (*P* < 0.05) in ACRB birds in comparison to FG and SG groups (Fig. 11W).

Figure S2 shows differential bacterial abundance between ACRB and FG birds (Fig. S2A), ACRB and SG birds (Fig. S2B) and FG and SG birds (Fig. S2C) in IL-L. There were 27 genera in ACRB, including Clostridiaceae, *SMB53*, Clostridiales and Coriobacteriales, and 5 genera in FG birds, including *Bacilli*, *Tricibacter*, Lactobacillales, *Streptococcus* and Enterococcaceae in greater abundance between these two lines of chickens (Fig. S2A). Comparison between ACRB and SG lines show 34 taxa and 6 taxa, respectively, in greater abundance, with the most abundant taxa being Clostridiales, CW040, Molicutes and TM7_3 in ACRB and *Bacilli*, Enterobacteriaceae, *Escherichia*, Gammaproteobacteria in SG birds (Fig. S2B). The lowest number of differentially abundant taxa was seen between FG and SG birds (6 and 3, respectively), including Proteobacteria, *Escherichia*, Bacillales, Gammaproteobacteria, Enterobacteriaceae and *Streptococcus* in FG line, and Clostridiaceae, Firmicutes and *Klebsiella* in SG birds (Fig. S2C).

In contrast, only a few taxa showed differential abundance between lines in IL-M (Fig. S3). Comparison between ACRB and FG birds revealed 9 and 6 differentially abundant taxa, respectively, with Faecalibacterium, OD1, Alphaproteobacteria, Ricketsialles, Rhizobiales and *Bacilli* in ACRB and *Bacilli*, Lactobacillales, *Turicibacter*, *Lactobacillus*, *Streptococcus*, *Ralstonia*, and *Bacillus* in FG (Fig. S3A). Differentially abundant taxa of OD1, Alphaproteobacteria, Ricketsialles, Peptostreptococcaceaa and *Renibacterium* in ACRB and Streptohyta and *Cyanobacteria* in SG were detected in the comparison between ACRB and SG birds (Fig. S3B). Comparison between FG and SG birds showed only *Turicibacter* and Bacillales taxa in FG to be differentially abundant (Fig. S3C). In CE-L, 5 taxa in ACRB and 4 taxa in FG were differentially abundant when the LEfSe analysis was performed to compare ACRB with FG birds (Fig. S4A). The differentially abundant taxa in ACRB were Faecalibacterium, *Ruminococcus*, Lachnospiraceae, Lachnospira and Dorea, while Dehalobacteriacea, Alphaproteobacteria RF32, *Streptococcus* and Lactobacillales were in FG birds. *Streptococcus* in SG birds were differentially abundant in comparison to ACRB birds (Fig. S4B). Comparison between FG and SG birds revealed *Streptococcus* and Leuconostocaceae in FG and *Ruminococcus* in SG to be differentially abundant between these two lines (Fig. S4C). Differentially abundant taxa between chicken lines in CE-M are depicted in Fig. S5. There were 2 and 7 differentially abundant taxa in ACRB and FG lines, respectively, with ACRB showing higher abundance of *Candidatus Arthromitus* and OD1, lower abundance of Clostridiales, *Lactobacillus*, *Streptococcus*, Ruminococcaceae, Alphaproteobacteria and Dehalobacteriacea in comparison to FG birds (Fig. S5A). ACRB birds also showed higher abundance of Clostridiaceae, Eubacterium and Acinetobacter and OD1 in comparison to SG birds, that were characterized by higher differential abundance of Ruminococcaceae, Lactobacillales, Bifidobacteriaceae and *Delftia* (Fig. S5B). Four differentially abundant taxa were detected in comparison between FG and SG birds, with higher abundance of *Blautia*, Leuconostocaceae and *Streptococcus* in FG and *Eggerthella* in SG (Fig. S5C).

A correlation analysis between relatively abundant bacterial taxa and body weight revealed significant correlation in all tissues (Table 5). In IL-L, the taxa negatively correlated with body weight included *Clostridium* and UNCL in FG and SG birds while those positively correlated with body weight were LAR and *Turicibacter* in FG and SG and *SMB53* in ACRB birds. In IL-M, *Clostridium* was negatively correlated with body weight in FG birds whereas *Faecalibacterium* in ACRB, FG and SG, and [*Ruminococcus*] in FG were positively correlated with body weight. In CE-L, the taxa negatively correlated with body weight included *Blautia*, *Escherichia*, and *Klebsiella* in ACRB, FG, and SG birds, *Streptococcus* in FG and SG birds, and *SMB53* and *Turicibacter* in ACRB birds. The taxa positively correlated with body weight included *Faecalibacterium* in ACRB, FG, and SG birds, *Coprococcus*, *Sutterella*, and UNCL in FG and SG birds. In CE-M, the taxa negatively correlated with body weight included *Blautia* in ACRB and FG, *Escherichia* in ACRB, FG, and SG birds, and *Klebsiella* in FG and SG birds. Positively correlated taxa with body weight included *Faecalibacterium* and *Helicobacter* in ACRB, FG, and SG birds, [*Ruminococcus*] in FG and SG birds, *Butyricicoccus* in SG, *Candidatus Arthromitus* in ACRB, and *Oscillospira* in FG birds.

**Table 5:** Spearman correlation between body weight and relative abundant taxa at genus level for ileal (IL) and cecal (CE) luminal (L) and mucosal (M) samples

Taxon	IL-L			IL-M			CE-L			CE-M		
	**ACRB**	**FG**	**SG**	**ACRB**	**FG**	**SG**	**ACRB**	**FG**	**SG**	**ACRB**	**FG**	**SG**
*Blautia*							−0.63^**^	−0.53^***^	−0.43^**^	−0.65^***^	−0.42^***^	−0.24
*Butyricicoccus*							−0.16	−0.13	−0.05	0.34	0.25	0.49^**^
*Candidatus Arthromitus*	0.29	0.1	0.09	0.25	0.14	0.25				0.45^*^	0.08	0.17
*Clostridium*	−0.15	−0.37^**^	−0.36^*^	−0.26	−0.30^*^	−0.1	−0.39	−0.24	−0.3	−0.27	−0.17	−0.22
*Coprococcus*							0.38	0.67^***^	0.31^*^			
*Escherichia*	0.23	0.15	0.11				−0.75^***^	−0.77^***^	−0.81^***^	−0.51^*^	−0.66^***^	−0.74^***^
*Faecalibacterium*				0.55^*^	0.72^***^	0.71^***^	0.69^***^	0.40^**^	0.55^**^	0.58^**^	0.61^***^	0.62^***^
*Helicobacter*										0.52^*^	0.45^***^	0.55^***^
*Klebsiella*							−0.59^**^	−0.73^***^	−0.64^***^	−0.4	−0.56^***^	−0.63^***^
*Lactobacillus*	0.17	0.2	0.28	0.03	0.07	0.05	−0.26	−0.09	0.08	−0.05	0.16	0.25
LAR	0.44	0.58^***^	0.45^**^	0.27	0.19	0.06	0.14	0.04	−0.03	−0.09	0.25	−0.08
*Oscillospira*							−0.29	0.23	−0.1	0.2	0.41^**^	−0.04
*Ruminococcus*							0.01	0.01	−0.18	0.04	0.12	−0.09
*[Ruminococcus]*				0.26	0.54^***^	0.19	−0.16	0.19	0.05	−0.02	0.58^***^	0.35^*^
*SMB53*	0.02	0.40^**^	0.28	−0.26	0.21	0.28	−0.64^*^	−0.19	−0.26			
*Streptococcus*	0.18	0.05	−0.13				−0.18	−0.34^**^	−0.37^*^			
*Sutterella*							0.43	0.42^***^	0.39^*^			
*Turicibacter*	0.12	0.26^*^	0.32^*^	−0.04	0.17	0.21	−0.61^**^	−0.23	−0.29			
UNCL	−0.31	−0.71^***^	−0.75^***^	−0.27	−0.05	−0.09	0.4	0.47^***^	0.47^**^	−0.43	0.02	−0.01

Values in the table represent correlation coefficient. Bold values represent significant positive and negative correlations

*ACRB* Athens Canadian Random Bred, *FG* fast growing chickens, *SG* slow growing chickens

Asterisks denote significant correlations (^*^0.01 < *P* < 0.05, ^**^0.001 < *P* ≤ 0.01*,* ^***^P ≤ 0.0001)

### Differences in predicted function of microbiota between lines

**Fig. 12:**
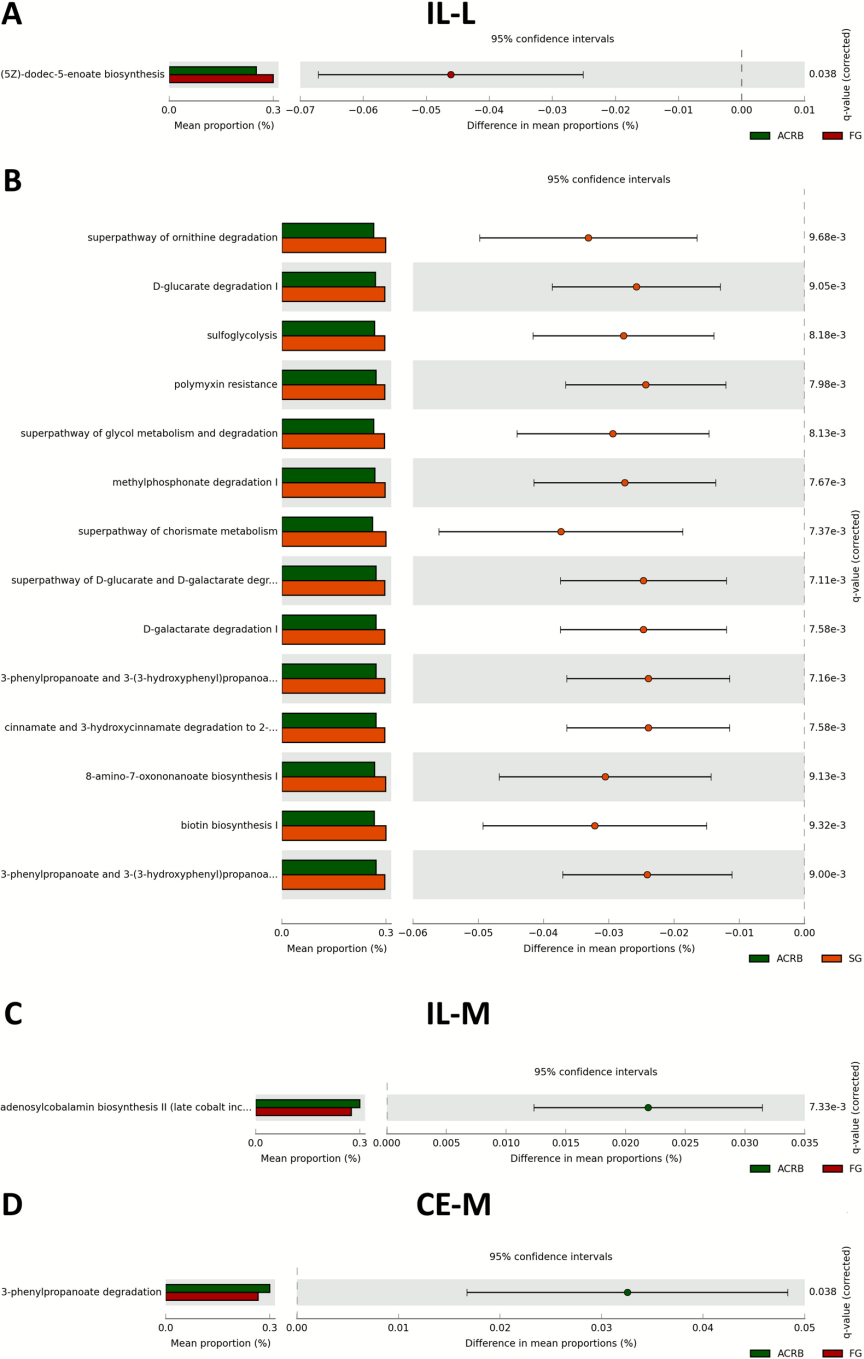
Effects of the line on predicted function of the ileal luminal (IL-L: **A** and **B**), (**C**) ileal mucosal (IL-M), and (**D**) cecal mucosal (CE-M) microbiota in chickens. Function of the microbiota was determined using PICRUST and visualized using STAMP. ACRB: Athens Canadian Random Bred; FG: Fast growing chickens; SG: Slow growing chickens

Principal component analysis of predicted function of microbiota in IL-L among three chicken lines revealed no evident clustering of the functions due to the line (Fig. S6A), while comparison between lines showed changes in relative abundance of genes for one pathway in ACRB vs. FG birds (Fig. 12A) and fourteen pathways in ACRB vs. SG birds (Fig. 12B). No significant differences in predicted function were observed for FG vs. SG comparison (data not shown). Specifically, ACRB birds were characterized by a decrease in relative abundance of pathway (5Z)-dodec-5-enoate biosynthesis in comparison to FG birds (Fig. 12A). Comparison between ACRB and SG lines showed an increase of metabolic pathways in SG in comparison to ACRB birds, with superpathway or ornithine degradation, D-glucarate degradation, sulfoglycolysis, polymyxin resistance and superpathway of glycol metabolism and degradation as the top pathways (Fig. 12B).

Similar to IL-L, no evident clustering of predicted function was observed due to the line in IL-M (Fig. S6B). In IL-M, there is only one pathway, adenosylcobalamin biosynthesis II, with higher relative abundance in ACRB birds in comparison to FG birds (Fig. 12C). No clustering of predicted metabolic pathways due to line of birds was observed in CE-L (Fig. S7A). Additionally, there were no significant (*P* > 0.05) differences in relative abundance of metabolic pathways between ACRB and FG, ACRB and SG, and FG and SG. Similar results were observed in CE-M (Fig. S7B), with only one metabolic pathway showing significant (*P* < 0.05) relative abundance between ACRB and FG birds (Fig. 12D). Metabolic pathway of 3-phenylpropanoate degradation showed higher relative abundance in ACRB birds in comparison to FG birds (Fig. 12D).

## Discussion

Modern chicken lines have been developed to increase meat production and reach market faster than slow and medium growing chickens. The increase in production performance is mainly due to genetic selection and is attributed to more efficient nutrient utilization. In the current study, the 3 lines (ACRB, SG, and FG) had similar weight at hatch and the first week post-hatch; however, from d 13 to 25, FG birds were characterized by the highest body weight while ACRB had the lowest body weight. In addition, FG and SG had lower FCR compared to ACRB birds. These results are consistent with previous data [5, 36, 37] and suggest that FG and SG chickens have better feed utilization compared to the heritage line ACRB. It has been hypothesized that the improvement in feed efficiency and growth is due to reduced maintenance energy in the intestine because selection has resulted in the reduction of relative weight of both the intestinal and mucosa and the improved feed utilization [6].

Recent data suggest that the improvement in growth performance could partly be attributed to the presence of intestinal microbiota [38, 39]. To determine if genetic selection for growth has affected the intestinal microbiota, ileal and cecal microbiota were studied in heritage and FG and SG chickens. It was believed that the chicken embryonic intestine was sterile; however, recent data suggest that the embryo inherits maternal microbiota during the laying process [40, 41]. In the modern poultry industry, there is no direct contact of hens with eggs and hatchlings don’t have a direct transfer of microbiome; therefore, the intestinal microbiome during incubation and early post-hatch are derived from the direct environment including incubators, hatchers, handlers, and feed. This suggests that intestinal microbiota may be influenced by hatchery provenance [42]. Because early exposure may influence future microbiota diversity [43], all chicken lines were hatched in house using same incubators and hatchers to remove all confounding factors on intestinal microbiota.

In the current study, no line effects were observed for alpha diversity differences in ileal and cecal content at 48 h before hatch and at hatch; however, some differences were observed due to time and line interaction. These results suggest that selection may have induced some microbiota differences over time in the cecum during incubation and at hatch. In term of early colonization of intestinal microbiota, changes were observed with greater relative abundance of the genus *Turicibacter* and lower relative abundance of species C*lostridium celatum* and LAR at −48 h compared to hatch suggesting a dynamic change in the establishment of intestinal microbiota during late embryonic stage and hatch.

The post-hatch period was marked by significant differences in alpha and beta diversity in the ileum and cecum. In the IL-L, alpha diversity indices were affected by time × line interaction, which was mainly driven by time because specific line differences were not seen at each time point. However, in IL-M, variations in alpha diversity metrices (richness and ASVs) were observed between ACRB and FG at d 14 post-hatch and SG birds at d 7 post-hatch, and this is most likely due to selection since the heritage birds exhibited higher number of unique and more abundant ASVs in agreement with a previous study on native chicken breeds and a modern broiler line [44]. It appears that differences observed during late embryonic development and hatch persisted during the post-hatch period, especially on d 7 and 14 post-hatch.

Contrary to the ileum, alpha diversity metrices (Richness, ASVs, and Shannon) in the cecum were mostly higher in FG compared to ACRB chickens at d 35 post-hatch. The reason why alpha diversity was increased in cecum vs. ileum is unclear. The cecum contains more bacteria than the other sections of the intestine [45] and is the site where most fermentation occur and energy is harvested [9]. At d 35 post-hatch, the intestinal microbiome has been established and the higher alpha diversity observed in the cecum of FG birds may be induced by selection for growth since higher alpha diversity has been linked to bigger birds [46], and this could partially explain weight differences between the ACRB and modern lines. These results agreed with previous reports where ACRB and jungle fowl lines had lower ASVs, and Shannon diversity compared to modern Random Bred 2015 line [47]. However, it seems that the ASVs were higher in ACRB than in the SG lines only at d 7 post-hatch. These differences could be explained by the dynamic change in intestinal microbiota early post-hatch since they disappear later during the post-hatch period when the intestinal microbiota may have been well established.

The beta diversity based on unweighted UniFrac (presence of species) was affected by time with low level overlaps between days and showing a shift in microbial diversity from d 7 to 21 and appearing to be stable by d 28 and d 35 post-hatch, especially in the cecum. More overlap was observed in ileal lumen and mucosa suggesting that the establishment and maturation of intestinal microbiota may differ by intestinal segment; however, this hypothesis needs to be further verified. These results are not surprising and confirm that intestinal microbiota in chickens is well established by the third week post-hatch [38, 48]. Contrary to time, no clear effect of line was observed for unweighted UniFrac, suggesting no effects of selection on the presence of bacterial species in the ileum and cecum. While no differences in species present were observed for line in the ileum and cecum, the abundance of genera and species may be affected by selection.

Genera *Streptococcus* and *Escherichia* are commensal bacteria of chicken gut, but they comprise pathogenic species that can cause major infection in humans and animals [49]. The increase of these genera in the IL-L of FG birds may make them more susceptible to infections should dysbiosis occur. The species *E. coli* was increased in SG compared to ACRB. Some *E. coli* strains may increase the risk of systemic infection in chickens [50] and significantly reduce production performance because the energy for growth would be used for the activation and the maintenance of the immune system [51]. The relative abundance of LAR species in IL-L was higher in ACRB compared to both FG and SG birds in the current study, suggesting an increase in diversity in IL-L of ACRB birds since we have observed increased ASVs and richness in ACRB at d 7 and 10 as previously mentioned. The increase in microbial diversity may be associated with improved gut health as previously hypothesized [16] and body weight in chickens [52].

There were significant correlations between body weight and the relative abundant taxa at genus level in intestinal segments used in the current study. It is worth noting that the correlations were not consistent across tissues (ileum and cecum) and sampling (luminal and mucosal). Overall, some bacterial taxa including *Coprococcus*, [*Ruminococcus*], and *Turicibacter* were positively correlated with body weight in FG and SG whereas *Clostridium*, *Streptococcus*, and UNCL showed a negative correlation. *Butyricicoccus* was positively correlated with body weight in only SG and *Candidatus Arthromitus* in ACRB. Interestingly, *SMB53* was positively correlated with body weight in FG (IL-L) but negatively correlated with body in ACRB (CE-L). The genus *SMB53* is common in animal intestine including chickens and swine. It belongs to the Clostridiaceae family and can degrade mucus and consume glucose [53, 54]. The reduction in the relative abundance of *SMB53* in IL-L of FG birds may be beneficial and help preserve nutrients compared to ACRB.

Other genera including *Turicibacter* [55] and *Ruminococcus* [56] may be involved in nutrient metabolism and fiber degradation, respectively. The genus *Turicibacter* comprises species that have bile salt hydrolase activities and could affect host lipid metabolism [55]. The reason for the increase of this genus in CE-M of FG line is unclear, but it could play an important role in fat deposition and lipid profile in FG birds; however, this hypothesis needs further investigations. Most species belonging to the *Ruminococcus* genus can degrade dietary fiber including cellulose, hemicellulose, and polysaccharides and provide nutrients for the host [56, 57]. The reduction in *Ruminococcus* in CE-M of FG compared to ACRB and SG lines suggests less degradation of dietary fiber, consequently reducing nutrients availability from bacterial fermentation in FG lines since lower *Ruminococcus torques*, as species of the genus Ruminococcus, has been correlated with lower short chain fatty acid production [58]. In contrast, *Ruminococcus torques* was increased in high market weight compared to low market weight chickens [59]. In the previous study [59], the two groups of chickens were selected from the same genetic line, and this could partly explain the discrepancy. Although the increased abundance of *Ruminococcus torques* may provide nutrients, they can also degrade mucin [60] thereby reducing intestinal integrity.

The relative abundance of *Lactobacillus salivarius* was higher in IL-M of FG compared to ACRB and in both FG and SG compared to ACRB in CE-L. Some species such as *L. salivarius* can produce bacteriocins against pathogenic bacteria and are good probiotic candidates in the poultry industry [61]. It has been shown to improve growth performance and immune response in broilers challenged with *E. coli* [62, 63]. The increased relative abundance of *L. salivarius* in IL-M of FG birds is a candidate contributor to the observed performance difference, however this hypothesis requires future validation.

Interestingly, the relative abundance of the genus *Candidatus Arthromitus* was higher in CE-M of ACRB compared to FG birds while being intermediate in SG birds. *Candidatus Arthromitus* is a commensal bacteria found in most animal intestines, including poultry [64, 65] and fish [66]. It is a segmented filamentous bacteria belonging to the Clostridial family and can penetrate the mucus layer and attach to the intestinal wall without invading epithelial cells. *Candidatus Arthromitus* is known for its effects on the maturation of the host innate and adaptive immune system [64]. The increase in relative abundance of *Candidatus Arthromitus* observed in the current study is likely to improve the immune response and intestinal health in ACRB bird since the *Candidatus Arthromitus* has been linked to beneficial impacts on the immune system [67]. In addition, this genus has been positively correlated with body weight only in ACRB in the current study. It can be assumed that selection for improved growth rate has resulted in the reduction of *Candidatus Arthromitus*, and this may partly explain the reduced intestinal health in modern broiler chickens; however, this hypothesis needs to be further explored since the use of *Candidatus Arthromitus* as probiotics is rare in broiler chickens [39].

To determine the effect of genetic selection on intestinal microbiota, we also performed a pairwise differential abundance analysis between the three lines. Overall, in IL-L and IL-M, ACRB showed higher number of bacterial taxa compared to SG and FG. As previously mentioned, this may likely improve intestinal health in heritage compared to modern birds. However, the comparison between FG and SG birds showed that *Turicibacter* and Bacillales were differentially abundant only in FG birds. The abundance of *Turicibacter* in FG birds was mentioned in a previous paragraph. The order Bacillales comprises genus *Bacillus* and species that have been shown to improve feed efficiency and growth performance in broiler chickens [68] and this may be the cause of the growth performance differences observed between FG and SG birds. However, this hypothesis requires further confirmation. Because this order is abundant only in FG birds, it suggests that the degree of selection may have more impact on intestinal microbiota. Contrary to IL, CE microbiota showed less differentially abundant taxa as opposed to the ileum.

Commensal or pathogenic bacteria in the intestine use dietary nutrients through metabolic pathways to generate metabolites that can have direct effects on the host [69]. To determine potential changes in metabolic pathways associated with differential abundant taxa among ACRB, FG, and SG lines, we performed differential abundance analysis. In the IL-L, the predicted (5Z)-dodec-5-enoate biosynthesis pathway, which is involved in fatty acid synthesis, was increased in FG compared to ACRB birds suggesting that taxa involved in this pathway may provide additional nutrients for FG birds. Several predicted pathways were observed between ACRB and SG birds. Pathways related to the degradation of carbohydrates including D-glucarate and D-galactarate, and sulfoglycolysis were increased in SG compared to ACRB birds. D-Glucarate and D-galactarate are sugar acids that serve as substrates for *E. coli* [70] confirming the increased relative abundance of this bacterial species in SG birds.

Sulfoglycolysis allows bacterial degradation of sulfo-sugar sulfoquinovose [71]. The intermediary products of sulfoglycolysis may enter glycolysis [72], but other end products including butyrate have been reported in mice [73]. This suggest that the increase of sulfoglycolysis may benefit SG birds since butyrate is a key energy source for enterocytes [74].

In addition to degradation pathways, biosynthesis pathways include 8-amino-7-oxononanoate, which is the first step limiting enzyme involved in biotin synthesis. The predicted biotin biosynthesis pathway was higher in SG compared to ACRB birds. Besides dietary biotin sources, bacterial biotin production contributes substantially to host biotin levels [75]. It is possible that because SG birds are growing faster and heavier than ACRB birds, more biotin is needed to support cellular processes as well as carbohydrate, protein, and lipid metabolism [76]. Similar patterns were observed with the superpathway of chorismate metabolism, which is important for aromatic amino acid synthesis. In addition, the superpathway of ornithine degradation was increased. This pathway is involved in the production of polyamines, which are essential for intestinal epithelial cell proliferation and intestinal barrier integrity. This may indicate that more bacterial polyamides may be available, enhancing intestinal function in SG birds [77]. Although we have speculated that the functional predictions mentioned above in bacteria may benefit the host, the predictive functional analysis can have limited accuracy outside of human studies and more investigation is needed to confirm those benefits in chickens.

It is worth mentioning some limitations to this study. The study was limited to 35 d while the modern broiler’s market age is around 42 d, and only the ileum and the cecum were included in microbiome analysis. Future studies should include the entire production cycle to capture microbiota profile at the time of harvest and all intestinal segments.

## Conclusion

Modern broiler chickens have been selected to improve feed efficiency and growth rate and therefore harvesting more nutrients and energy from the diet suggesting that they may have different intestinal microbiota profile. The results from this study indicate that selection has affected intestinal microbiota as bacterial diversity was different in the ileum and cecum. While diversity was increased in the ileum mucosa at early (d 7 and 14) during the post-hatch in ACRB lines, it increased in FG birds later (d 35) post-hatch. Differences in bacteria abundance appear to be specific to genetic line, with some bacteria responsible for producing metabolites that would benefit modern broilers. Predicted metabolic pathways suggest that more pathways related to biosynthesis of nucleotides and biotin, especially in the ileum, were likely increased in the light of higher growth rate in SG compared to ACRB lines. Although these data have improved our understanding of the impacts of selection on intestinal microbiota, the direct effects of changes in microbiota and predicted functions on the host metabolism and nutritional status that explain, at least partly, the production efficiency in modern broiler lines are still unknown.

## Supplementary Information

Additional file 1: Table S1. PERMANOVA *P*-values for interactive or main effects for bacterial community beta diversity in ileal and cecal luminal and mucosal samples. Fig. S1. Taxonomic profile [relative abundance] in ileal and cecal bacterial populations in embryos and chicks at hatch and linear discriminant analysis effect size in comparison of ileal microbiota in embryos and chicks at hatch. Fig. S2. Comparison between lines of differentially abundant bacterial taxa as determined by linear discriminant analysis effect size in ileal luminal microbiota. Fig. S3. Comparison between lines of differentially abundant bacterial taxa as determined by linear discriminant analysis effect size in ileal mucosal microbiota. Fig. S4. Comparison between lines of differentially abundant bacterial taxa as determined by linear discriminant analysis effect size in cecal luminal microbiota. Fig. S5. Comparison between lines of differentially abundant bacterial taxa as determined by linear discriminant analysis effect size in cecal mucosal microbiota. Fig. S6. Effect of the line on predicted function of the ileal luminal and mucosal microbiota in chickens. Fig. S7. Effect of the line on predicted function of the cecal luminal and mucosal microbiota in chickens.

## Data Availability

All relevant data are within the paper. The 16S rRNA gene sequences were deposited in the NCBI Sequence Read Archive PRJNA1280974).

## Abbreviations

ACRB: Athens Canadian Random Bred
ASVs: Amplicon sequence variants
CE: Cecal
CE-L: Cecal lumen
CE-M: Cecal mucosa
DNA: Desoxyribonucleic acid
FG: Fast growing
LEfSe: Linear discriminant analysis (LDA) Effect Size
IL: Ileal
IL-L: Ileal lumen
IL-M: Ileal mucosa
LAR: Low abundance reads
MAFFT: Multiple Alignment using Fast Fourier Transform
PCoA: Principal coordinate analysis
PERMANOVA: Non-parametric permutational analysis of variance
PICRUSt: Phylogenetic Investigation of Communities by Reconstruction of Unobserved States
PCR: Polymerase chain reaction
QIIME: Quantitative Insight Into Microbial Ecology
SG: Slow growing
SRA: Sequence Read Archive
