# Supplementary material for: Effects of selection on production parameters and intestinal microbiota in heritage and modern broiler chickens
Source: J Anim Sci Biotechnol. 2026 Mar 7;17:40. doi: 10.1186/s40104-026-01360-8 (PMC12967013; doi:10.1186/s40104-026-01360-8)
Supplement: Supplementary file 1 — Additional file 1: Table S1. PERMANOVA P-values for interactive or main effects for bacterial community beta diversity in ileal and cecal luminal and mucosal samples. Fig. S1. Taxonomic profile [relative abundance] in ileal and cecal bacterial populations in embryos and chicks at hatch and linear discriminant analysis effect size in comparison of ileal microbiota in embryos and chicks at hatch. Fig. S2. Comparison between lines of differentially abundant bacterial taxa as determined by linear discriminant analysis effect size in ileal luminal microbiota. Fig. S3. Comparison between lines of differentially abundant bacterial taxa as determined by linear discriminant analysis effect size in ileal mucosal microbiota. Fig. S4. Comparison between lines of differentially abundant bacterial taxa as determined by linear discriminant analysis effect size in cecal luminal microbiota. Fig. S5. Comparison between lines of differentially abundant bacterial taxa as determined by linear discriminant analysis effect size in cecal mucosal microbiota. Fig. S6. Effect of the line on predicted function of the ileal luminal and mucosal microbiota in chickens. Fig. S7. Effect of the line on predicted function of the cecal luminal and mucosal microbiota in chickens. [file 40104_2026_1360_MOESM1_ESM.docx]

Table S1. PERMANOVA p-values for interactive (Time x Line) or main (Time, Line) effects for bacterial community beta diversity (PERMANOVA results) in ileal (IL) and cecal (CE) luminal (L) and mucosal (M) samples collected from days 7 to 35 post-hatch.

|  | Pr = F | | | |
| --- | --- | --- | --- | --- |
|  | IL-L | IL-M | CE-L | CE-M |
| Unweighted UniFrac Distance | | | | |
| Time | 0.001 | 0.001 | 0.001 | 0.001 |
| Line | 0.015 | 0.095 | 0.674 | 0.134 |
| Time×Line | 0.001 | 0.001 | 0.001 | 0.001 |


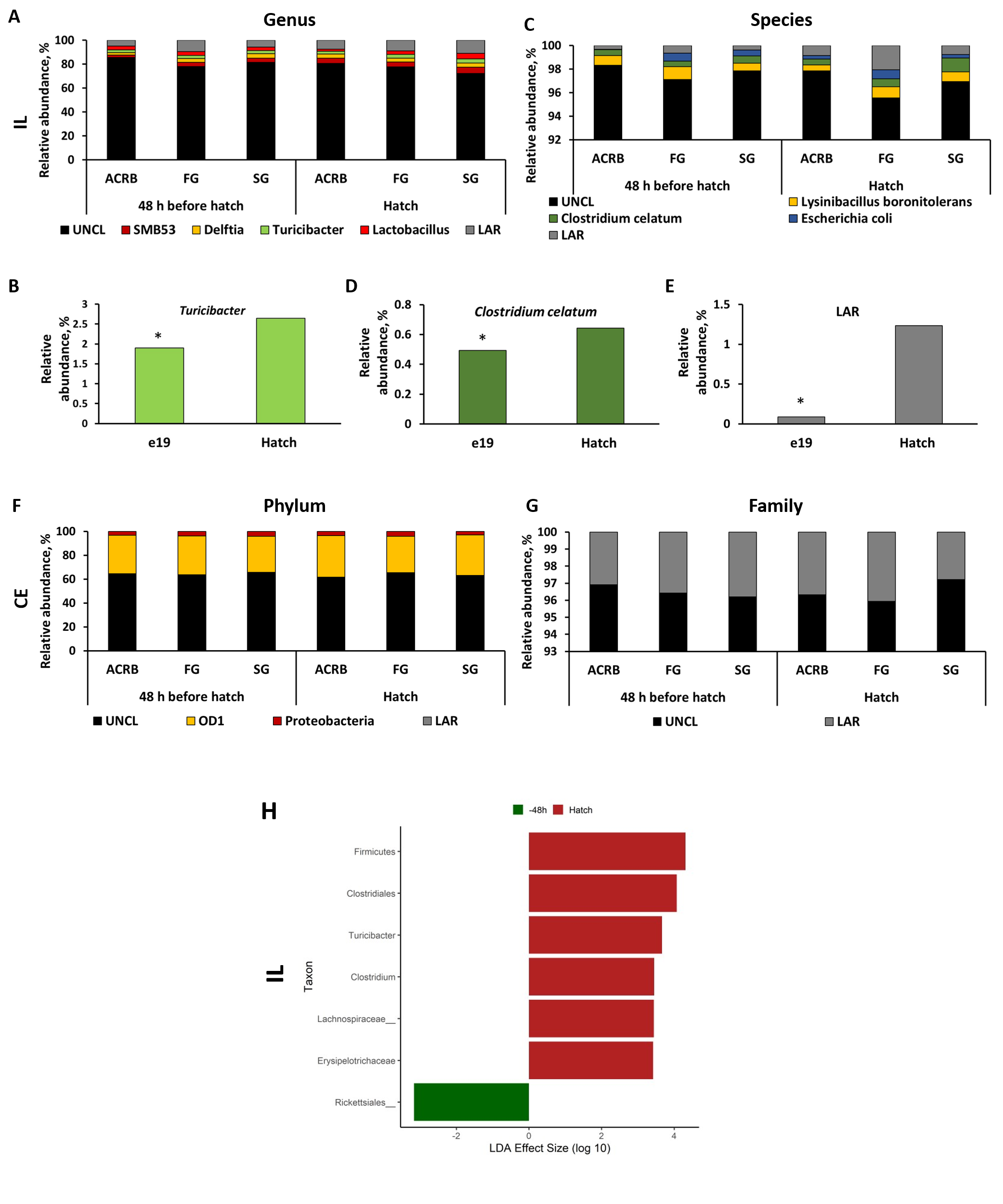


**Fig. S1.** Taxonomic profile [relative abundance (%)] in ileal (IL) and cecal (CE) bacterial populations in embryo (48 h before hatch) and chicks at hatch. Taxonomic profile of chicken IL at (A) genus and (C) species level. Effect of time (-48 h before hatch and at hatch) on (B) *Turicibacter,* (D) *Clostridium celatum,* and (E) low abundance reads (LAR) level in IL. Taxonomic profile of chicken CE at (F) phylum and (G) family level. Asterisks denote statistically significant (*P*<0.05) differences between 48 h before hatch and hatch chickens.

Linear Discriminant analysis (LDA) effect size (LEfSe) in comparison of ileal (IL) microbiota (H) in embryos (-48 h, e19) and chicks at hatch. Positive effect size indicates higher relative abundance in chicks at hatch, while negative effect size indicates higher relative abundance in embryos 48 h before hatch. UNCL– unclassified bacterial reads, e19 (48h before hatch), ACRB – Athens Canadian Random Bred, FG – fast growing chickens, SG – slow growing chickens.


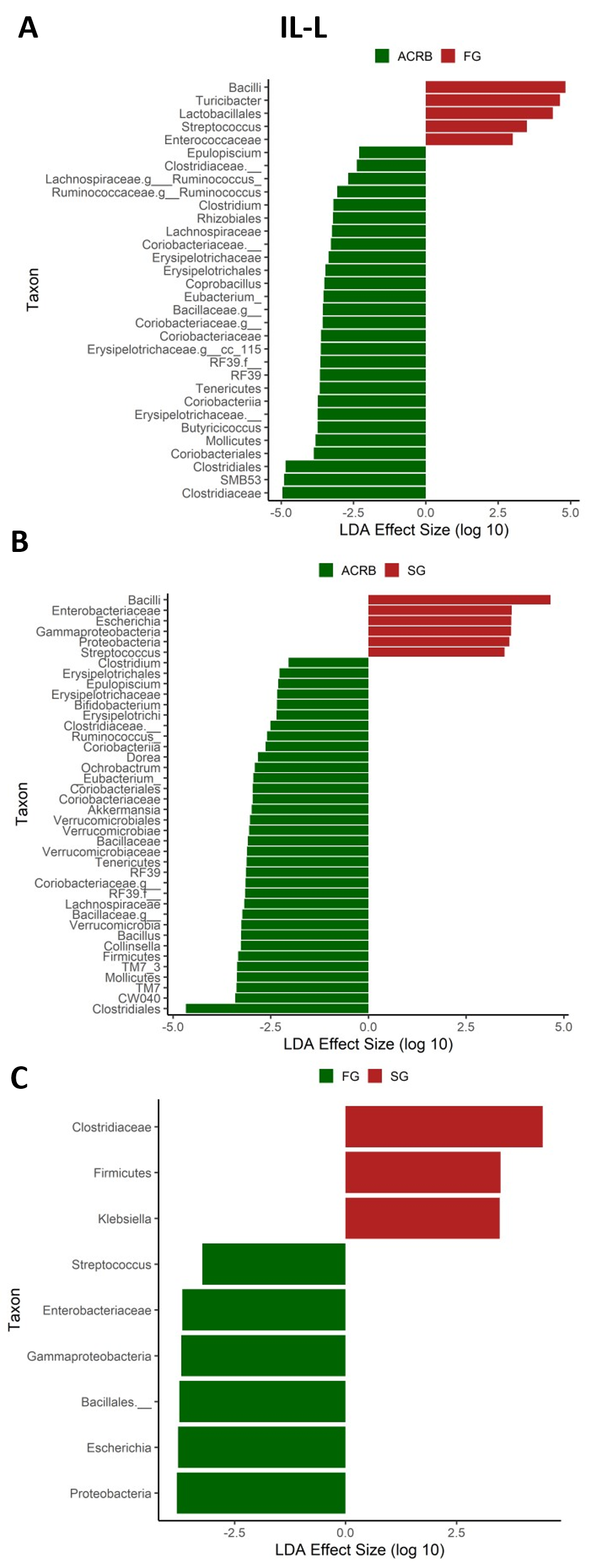


**Fig. S2.** Comparison between lines of differentially abundant bacterial taxa as determined by Linear Discriminant Analysis (LDA) effect size (LEfSe) analysis in ileal luminal (IL-L) microbiota. (A) comparison between ACRB and FG chickens with positive effect size indicates higher relative abundance in FG chickens while negative effect size indicates higher relative abundance in ACRB chickens, (B) comparison between ACRB and SG chickens with positive effect size indicates higher relative abundance in SG chickens while negative effect size indicates higher relative abundance in ACRB chickens, and (C) comparison between FG and SG chickens with positive effect size indicates higher relative abundance in SG chickens while negative effect size indicates higher relative abundance in FG chickens. ACRB – Athens Canadian Random Bred, FG – fast growing chickens, SG – slow growing chickens.


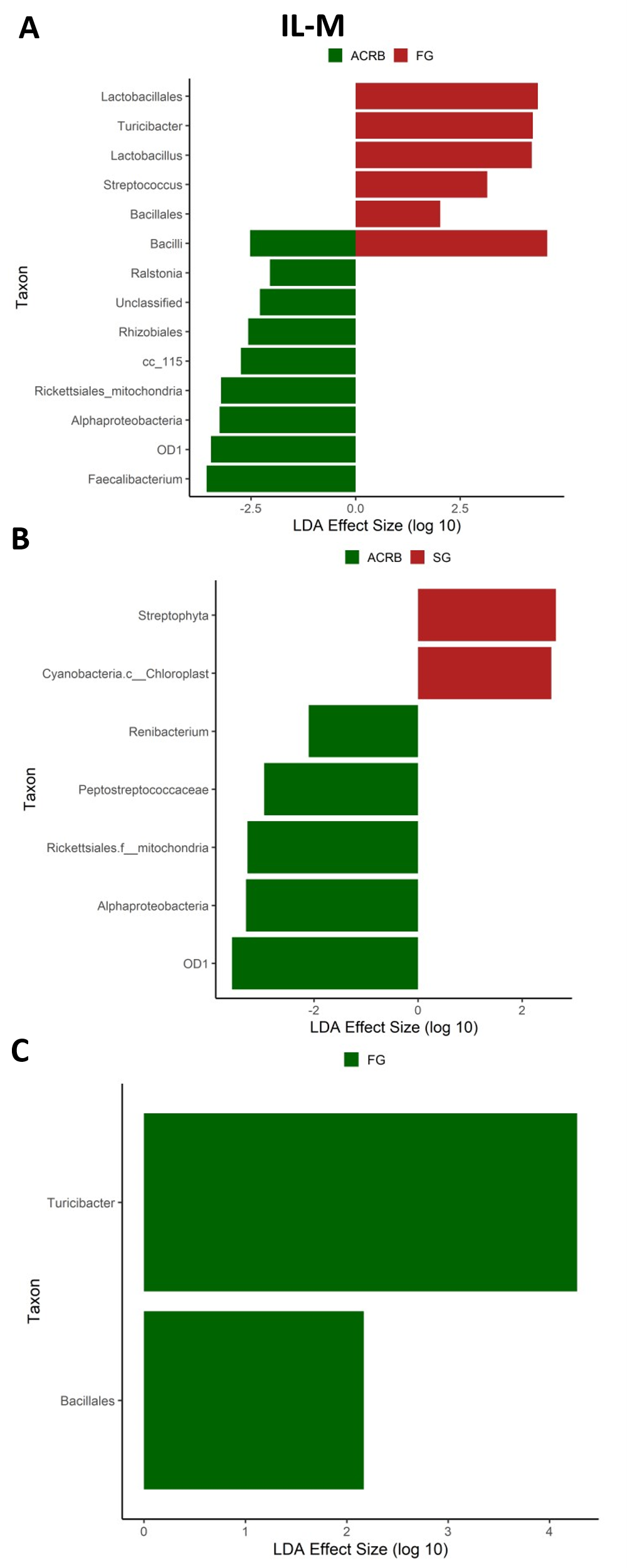


**Fig. S3.** Comparison between lines of differentially abundant bacterial taxa as determined by Linear Discriminant Analysis (LDA) effect size (LEfSe) analysis in ileal mucosal (IL-M) microbiota. (A) comparison between ACRB and FG chickens with positive effect size indicates higher relative abundance in FG chickens while negative effect size indicates higher relative abundance in ACRB chickens, (B) comparison between ACRB and SG chickens with positive effect size indicates higher relative abundance in SG chickens while negative effect size indicates higher relative abundance in ACRB chickens, and (C) comparison between FG and SG chickens with positive effect size indicates higher relative abundance in FG chickens while no differentially abundant taxa were determined in SG chickens. ACRB – Athens Canadian Random Bred, FG – fast growing chickens, SG – slow growing chickens.


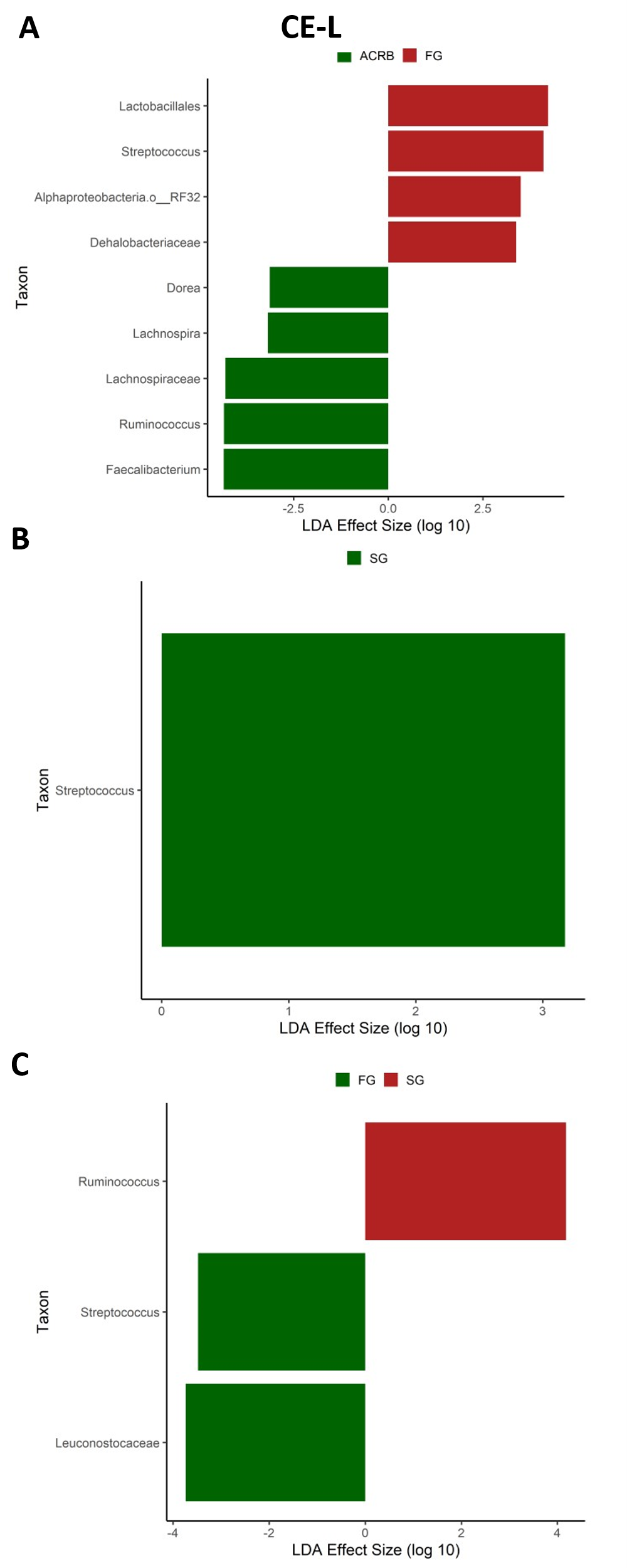


**Fig. S4.** Comparison between lines of differentially abundant bacterial taxa as determined by Linear Discriminant Analysis (LDA) effect size (LEfSe) analysis in cecal luminal (CE-L) microbiota. (A) comparison between ACRB and FG chickens with positive effect size indicates higher relative abundance in FG chickens while negative effect size indicates higher relative abundance in ACRB chickens, (B) comparison between ACRB and SG chickens with positive effect size indicates higher relative abundance in SG chickens while no differentially abundant taxa were determined in ACRB chickens, and (C) comparison between FG and SG chickens with positive effect size indicates higher relative abundance in SG chickens while negative effect size indicates higher relative abundance in FG chickens. ACRB – Athens Canadian Random Bred, FG – fast growing chickens, SG – slow growing chickens.


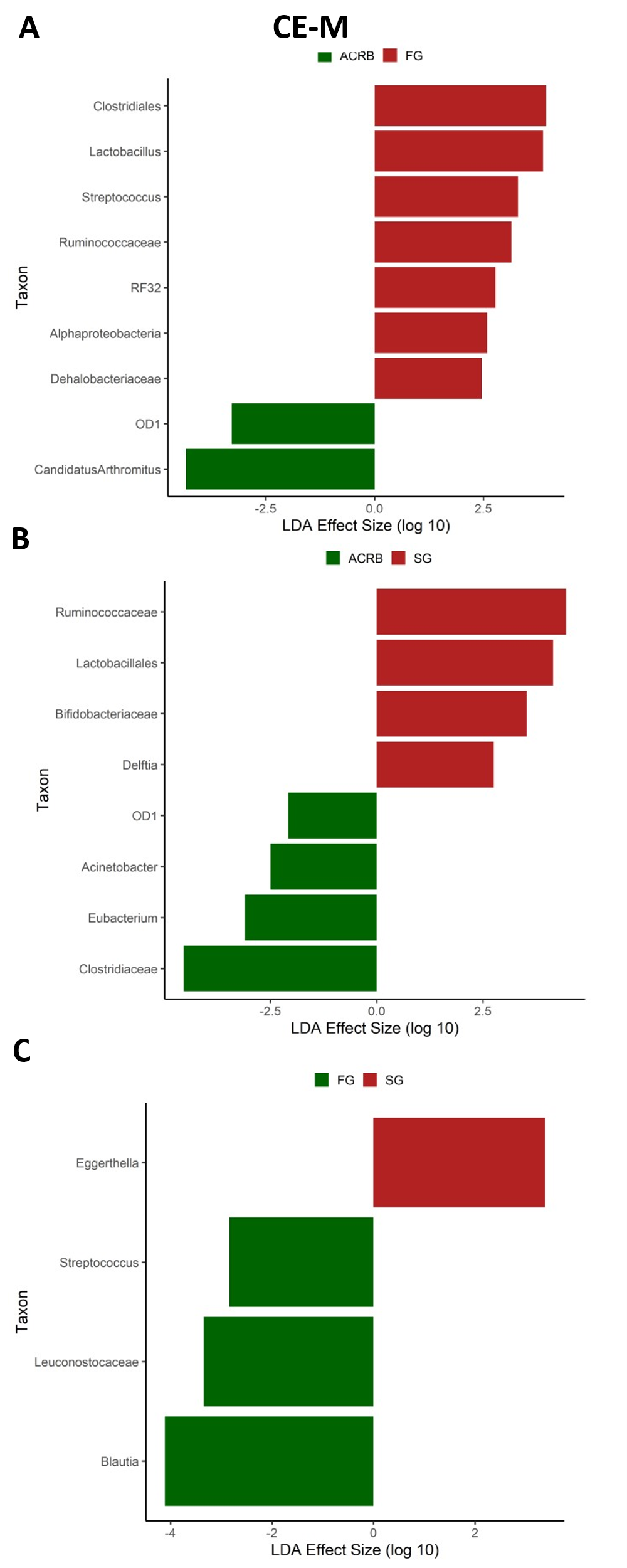


**Fig. S5.** Comparison between lines of differentially abundant bacterial taxa as determined by Linear Discriminant Analysis (LDA) effect size (LEfSe) analysis in cecal mucosal (CE-M) microbiota. (A) comparison between ACRB and FG chickens with positive effect size indicates higher relative abundance in FG chickens while negative effect size indicates higher relative abundance in ACRB chickens, (B) comparison between ACRB and SG chickens with positive effect size indicates higher relative abundance in SG chickens while while negative effect size indicates higher relative abundance in ACRB chickens, and (C) comparison between FG and SG chickens with positive effect size indicates higher relative abundance in SG chickens while negative effect size indicates higher relative abundance in FG chickens. ACRB – Athens Canadian Random Bred, FG – fast growing chickens, SG – slow growing chickens.


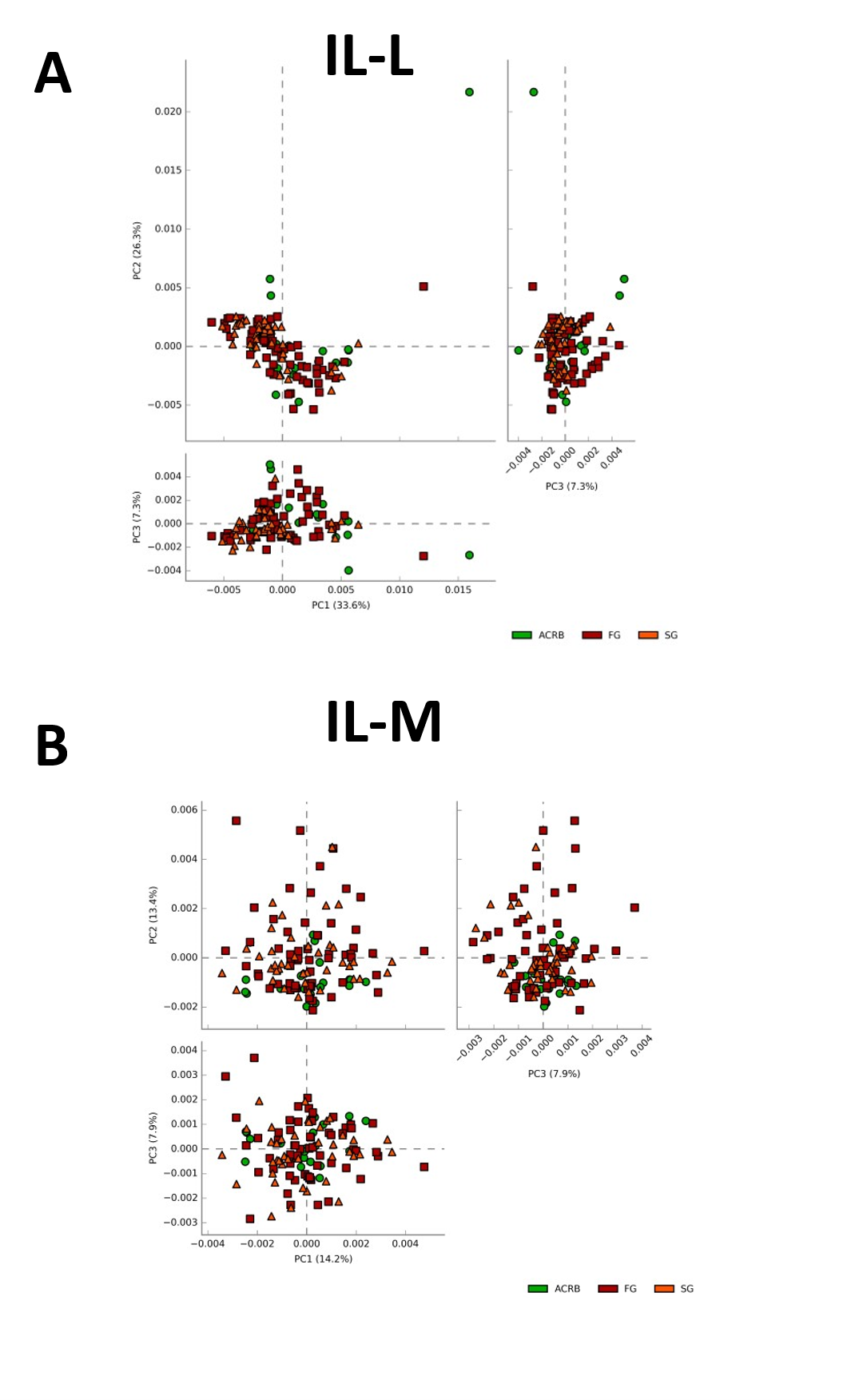


**Fig. S6.** Effect of the line on predicted function of (A) the ileal luminal (IL-L) and (B) the IL-mucosal (IL-M) microbiota in chickens. Function of the microbiota was determined using PICRUST and visualized using STAMP. ACRB – Athens Canadian Random Bred, FG – fast growing chickens, SG – slow growing chickens.


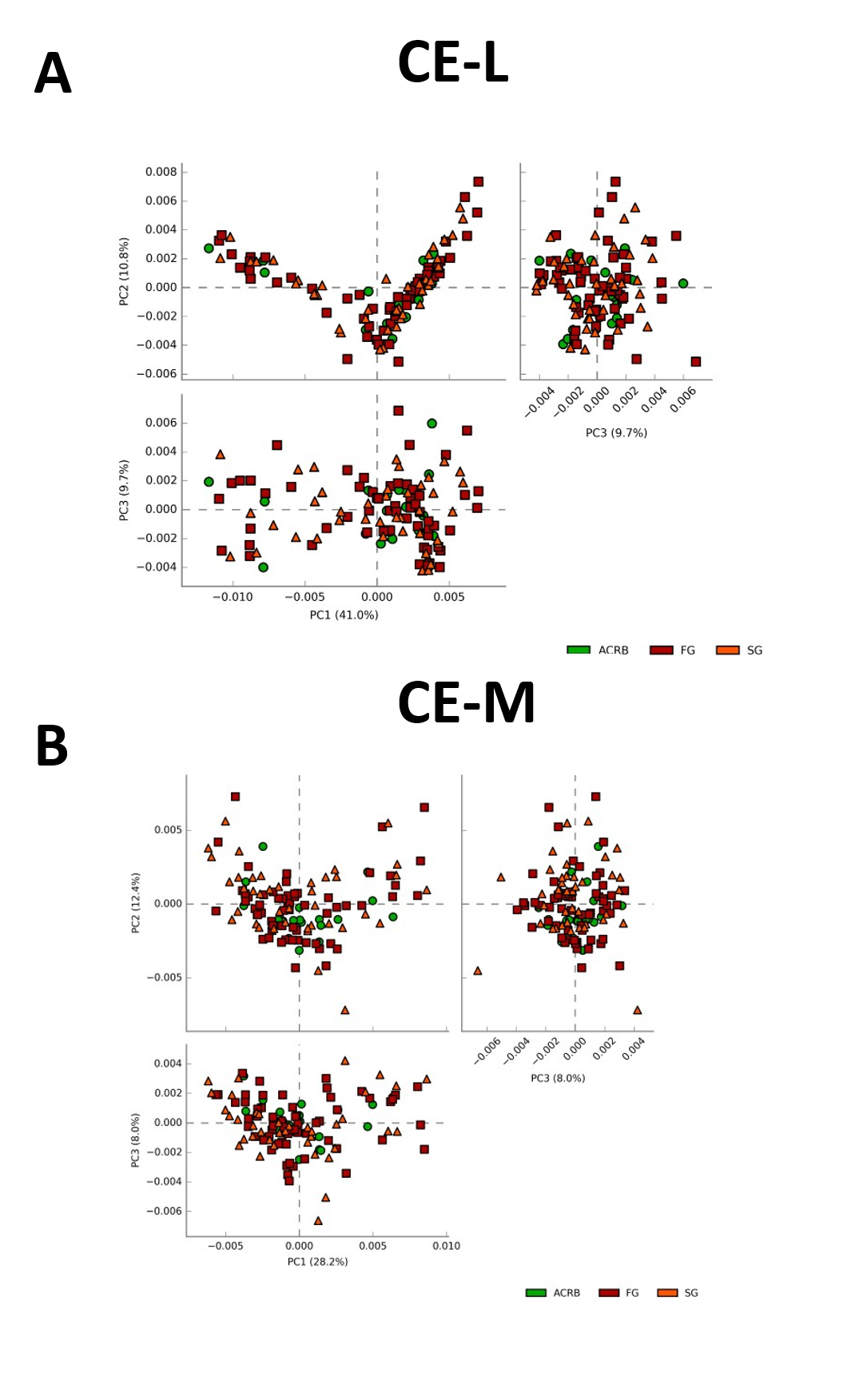


**Fig. S7.** Effect of the line on predicted function of (A) the cecal luminal (CE-L) and (B) the CE-mucosal (CE-M) microbiota in chickens. Function of the microbiota was determined using PICRUST and visualized using STAMP. ACRB – Athens Canadian Random Bred, FG – fast growing chickens, SG – slow growing chickens.
